# Species limits in *Veronica hederifolia* and relatives: experimental, morphometric, and phylogenetic approaches in a weedy polyploid complex

**DOI:** 10.1186/s12870-026-10212-6

**Published:** 2026-10-22

**Authors:** Dirk C. Albach, Pavel Kúr

**Affiliations:** 1 Institute for Biology and Environmental Sciences, Carl von Ossietzky- Universität Oldenburg, Oldenburg, Germany; 2 Jan Evangelista Purkyně University, Ústí nad Labem, Czechia

**Keywords:** Common garden experiment, DNA sequencing, Mediterranean, Morphometry, Phenotypic plasticity, Polyploidy, Triploid plants, *Veronica hederifolia*

## Abstract

**Background:**

Polyploid speciation gains increasingly more attention with the ease of larger scale analyses using flow cytometry. Therefore, the analysis of morphological differences discriminating different cytotypes gains increasing importance. *Veronica hederifolia* and relatives constitute such a group of species, easily differentiated by ploidy but with controversially discussed morphological differences. Here, we provide a comprehensive analysis of DNA sequence data, ploidy analysis and a morphometric study of herbarium material and a growth chamber experiment with variable light and water conditions to understand species differentiation in this group.

**Results:**

Our results support the recognition of three species in Central Europe differing in ploidy. Diploid *V. triloba* can be differentiated not only by ploidy but also by a low pedicel/calyx length ratio (< 1.59). Tetraploid *V. sublobata* can be differentiated from *V*. *hederifolia* not only by a longer pedicel/calyx ratio but more consistently by scattered hairs around the pedicel and shorter hairs on the calyx. Leaf characters are highly influenced by light and, therefore, not diagnostic at the species level. We also report the first triploid plant in the group but such hybrids seem to be rare.

**Conclusions:**

Based on these characters the three Central European species can be reliably differentiated. However, our data also stress the need to investigate the diversity of the group in the Mediterranean more intensively since here the phylogenetic and morphological variation has not been sufficiently analyzed up to now.

**Supplementary Information:**

The online version contains supplementary material available at https://doi.org/10.1186/s12870-026-10212-6.

## Background

Polyploidization is a common process in angiosperms. Ever since its discovery, researchers have sought to understand the reasons for its success and the conditions that promote its establishment and spread [1, 2]. More complex scenarios arise in polyploid taxa in which ancestor and progenitor lineages coexist sympatrically, sometimes within mixed populations. Here, dense sampling is necessary to determine cytotype-specific distribution areas, migration routes, and the frequency of independent polyploid origins.

Polyploidy is especially common in the genus *Veronica*, in which 25% of speciation events were inferred to be associated with polyploidy [3]. One intriguing case for diversification of polyploids in the genus is *Veronica* subsect. *Cochlidiosperma* with three cytotypes. Here, diploids are predominantly found in southern parts of Europe, and tetra- and hexaploids are widespread in Western and Central Europe [4]. Such taxa with three cytotypes are remarkably common in Europe and the Mediterranean and even in the sister group, *V*. subsect. *Cymbalariae*, in which multiple origins of the tetra- and hexaploids were inferred [5].

*Veronica* subsect. *Cochlidiosperma* comprises up to five currently recognized species of sprawling annuals, widely distributed across Europe and southwest Asia [4]. Variation within this subsection is influenced by factors that commonly obscure species boundaries: phenotypic plasticity, a selfing breeding system, short generation times, hybridization accompanied by polyploidy [6], and a broad ecological range—from the Mediterranean to Scandinavia [7], with the greatest phenotypic diversity in the Mediterranean. Due to their strong association with anthropogenically disturbed habitats, it remains uncertain to what extent their current distribution—particularly within Europe—is natural or human-mediated. However, human activity has clearly facilitated the dispersal of three species to North America, Eastern Asia, Australia, and New Zealand [8] with limited taxonomic consequences. Of the five species, *V*. *hederifolia* is the only one originally recognized by Linné [9], with all other species subsequently split from it. The high variability within the group has led to the publication of approximately 100 names, many of which are obscure or poorly defined.

Linné [9] recognized *V*. *hederifolia* as the only species of the subsection as circumscribed today, but doubts about that arose soon after with the description of *V*. *lappago* [10] based on specimens with more acute and deeply divided leaves. This is now considered a modification of *V*. *hederifolia* (as supported by our investigations below), and leaf shape is not considered a useful character for distinguishing the species. Subsequently, Opiz [11] published *V*. *hederifolia* var. *triloba*, which was recognized at specific rank soon after [12]. However, this view was not generally accepted due to the large variation in *V*. *hederifolia* [13] until a more detailed analysis by Lehmann [14]. Subsequently this was supported by ploidy analyses that showed *V*. *triloba* is diploid and *V*. *hederifolia* hexaploid [3].

With regard to the tetraploids in *V.* subsect. *Cochlidiosperma*, Klett [15] published the combination *V*. *hederifolia* var. *lucorum*, which is nowadays considered a synonym of the tetraploid *V*. *sublobata*. Lange [16] noted among plants from Denmark that on cultivated fields the leaves are larger and fleshy; in shade (*V*. *hederifolia* var. *umbrosa* Mort.) leaves are smaller, ovate, three-lobed, the stem is more erect, hairy on all sides, flowers smaller and stamens paler – characters used to distinguish *V*. *sublobata* from *V*. *hederifolia*. However, this species was only much later recognized at the species level [6] and discussions around its validity continue to the present day [4, 17–23]. Various characters were used to distinguish the two species (Table 1, Suppl. Table 1). Fischer [6] initially differentiated *V*. *sublobata* based on ploidy level and a combination of leaf length, leaf incision, pedicel length in fruit, calyx length and average pollen diameter, while also highlighting flower color and mentioning a number of other characters such as calyx hair length, pedicel indumentum, and style length (Suppl. Table 1), but these differences were often disputed.

**Table 1:** Currently recognized taxa in *V*. subsect. *Cochlidiosperma* and their main flower characters and chromosome numbers

Species	Corolla size (diameter in mm)	Corolla lobe color	Nectar guides	Corolla tube color	Anther color	Style length (in mm)	Chromosome number (ploidy)
*V. stewartii*	2–3	White	Absent	White	?	0.2–0.3	2n = 18 (2x)
*V. sibthorpioides*	2–4	Pale blue	Slightly darker	White	White	0.2–0.6	2n = 28, 30 (4x)
*V. triloba*	3–6	Deep blue	Slightly darker	White	Dark blue	0.5–0.9	2n = 18 (2x)
*V. sublobata*	3–5(-6)	Pale violet to pale pink	Indistinct	Pale pink to white	White to pale violet	0.3–0.6	2n = 36 (4x)
*V. hederifolia*	5–7(-9)	Bright to pale blue	Darker	White	Sky blue	0.7–0.9	2n = 54 (6x)

All members of *V*. subsect. *Cochlidiosperma* are annuals, likely winter annuals, germinating under mild, wet conditions typical of Mediterranean climates [24]. They flower early in spring and set seed before the onset of summer drought [25]. This strong selection for rapid development and early reproduction has led to a reduction in vegetative structures in favor of reproductive ones. The vegetative part often constitutes less than 5% of the plant’s total length. The limited photosynthetic capacity of the vegetative parts is compensated for by enlarged, photosynthetically active reproductive structures. Especially, the bracts subtending each flower are enlarged and leaf-like. (In this paper, we will, for simplicity, not refer to them as bracts despite the fact that this is the right term for these structures.)

Nonetheless, leaf size and shape also show significant variation, only partially attributable to genetic factors. Phenotypic plasticity accounts for the remainder. For instance, individuals of *V*. *hederifolia* and *V*. *sublobata* can range from dwarf forms (5 cm) to robust forms exceeding 50 cm under nutrient-rich, well-watered conditions. Thus, controlled environment analyses are necessary to understand interactions between genotype and environment.

These evolutionary factors also favor selfing, a breeding system commonly associated with annual species in general [26, 27], although probably with variation among species and populations [6]. Despite the lack of clear investigations on the breeding system and the possibility that these early-flowering species attract a number of pollinators due to scarcity of competition in the early season, flower visits are rarely seen, and the high seed set suggests a high rate of selfing. The small flowers and short styles also suggest selfing based on the selfing syndrome in the genus [28], but with variation among species (Table 1).

To date, Albach [5] remains the only study to include all five species in a phylogenetic framework, though each was represented by a single sample. Analyses using plastid *trn*L-F sequences, nuclear ribosomal DNA (nrDNA), and AFLP fingerprints consistently identified the Asian species *V. stewartii* as the earliest diverging lineage. However, the three datasets yielded conflicting relationships among the remaining species. In particular, plastid DNA data supported a role of *V. sibthorpioides* in the origin of *V*. *sublobata* and a role of *V*. *triloba* in the origin of *V*. *hederifolia*. Nuclear data further supported *V*. *triloba* as a parental lineage of *V*. *hederifolia*. However, there is a disagreement between the ITS and AFLP datasets regarding the second parental lineage of *V*. *sublobata*. Thus, hybridization should be considered a possibility despite the apparent high selfing rate.

Given the unresolved species boundaries within this group, a more comprehensive investigation of genetic variation is warranted. Integrating these data with ploidy levels and morphological diversity will improve our understanding of the evolutionary processes shaping this subsection. To this end, we present the most detailed phylogenetic analysis of the group to date, alongside the most complete assessment of ploidy variation, including numerous new estimates derived from flow cytometry.

Our results demonstrate clear phylogenetic and cytotypic differentiation between *V*. *sublobata* and *V*. *hederifolia*. These findings prompted a growth chamber experiment on sympatric populations to identify diagnostic morphological characters that can reliably distinguish the two species. Additionally, our phylogenetic analysis uncovered several samples that did not cluster within the clades corresponding to currently recognized species. These outliers were examined in greater detail through morphological analysis of a broader set of herbarium specimens. Consequently, we here present a combination of data that demonstrate the way forward to understand the evolution of *V*. subsect. *Cochlidiosperma*: (1) A phylogenetic analysis of DNA sequence data that should form the basis for a future phylogenomic analysis, (2) a detailed flow cytometric analysis of a single, mixed-cytotype population demonstrating the variation in ploidy in such populations, of which we will need more in the future, and, finally, (3) a controlled-environment test of phenotypic variation for one genotype each, which needs to be expanded to understand phenotypic variation at the species level.

## Materials and methods

### Plant material

All plant material was initially assigned to species based on morphology [23, 29, 30]. If uncertain, specimens were assigned to species based on ploidy or leaf color (see below). Overall, we present data based on five different datasets, DNA sequencing, flow cytometry, growth chamber experiment, morphometry of plants from Czechia, and morphometry of plants from Türkiye. Voucher specimens were made for all datasets (Tables 2 and 3, Suppl. Tables 2–4).

**Table 2:** Information on samples used in the phylogenetic analysis; FCM – flow cytometry, * sample also used in the morphological analysis

Taxon	Identification number	Origin	Collector and collection number	Herbarium and herbarium number (if available)	GenBank number (ITS/trnLF)	Ploidy estimated by FCM
*V. hederifolia*	2320/344	Cyprus	Albach 136	WU	AY741524/ AF510425	
*V. hederifolia*	2318	Czech Republic	Kúr, P, 86 − 22	OLD09947	PZ959344/ PZ986715	
V. hederifolia *	2311	Czech Republic, Mnisek pod Brdy	Albach 1224	OLD00550	PZ959338/ PZ986708	
*V. hederifolia*	2315	Germany, Hessia: Oberhöchstadt, Sportgelände, TK 5817/112	Wittig, R., 8941	OLD05799	-/ PZ986712	
*V. hederifolia*	2314	Germany, Hessia: Oberursel, Maasgrundweiher 5717/344	Wittig, R.	OLD05765	PZ959341/ PZ986711	
V. hederifolia *	2313	Germany, Lower Saxony: Conneforde	Albach 1507	OLD05763	PZ959340/ PZ986710	6x
V. hederifolia *	2312	Germany, Sachsen, Dresden, Reichenbachstr./ Teplitzer Str.	Albach 1326	OLD00551	PZ959339/ PZ986709	6x
*V. hederifolia*	2319	Iran, Prov. Khorasan Tangehogol	Kukkonen 5749	WU	PZ959345/ PZ986716	
*V. hederifolia*	1739	Spain, Andalucia, Sierra de la Horconera	Escobar-Garcia & Vitek 1739	OLD06050, W	PZ959360/ -	
*V. hederifolia* (was *V. triloba*)	2336	Türkiye Prov. Corum Bogazkale	Ehrendorfer, 24.3.2002	WU	PZ959358/ PZ986732	
V. hederifolia *	2309	Türkiye, Antalya: Termessos	Albach & Köröklü, D6-2	OLD00546	PZ959336/ PZ986706	
V. hederifolia *	2310	Türkiye, Antalya: near Abdal Musa	Albach & Köröklü, D2-7a	OLD00547	PZ959337/ PZ986707	
*V. hederifolia*	2317	Türkiye, Antalya: Termessos	Albach, D6-1	OLD00486	PZ959343/ PZ986714	
*V. sibthorpioides*	550	Spain	Martinez Ortega 831	SALA	AY850099/ AY540876	
*V. stewartii*	404	Pakistan	Rechinger 30,780	WU	AY741525/ AY540875	
V. sublobata *	2324	Austria, Salzburg, Tennengau Hallein, Kuchl	Albach D. C., S857	OLD05846	PZ959348/ PZ986720	
*V. sublobata*	2322	Germany, Hessia: Burkhardsfelden	Albach 1506	OLD05762	- / PZ986718	
V. sublobata *	2325	Germany, Northrhine-Westphalia: Assinghausen	Albach 1942	OLD08471	PZ959349/ PZ986721	
*V. sublobata*	2316	Germany, Northrhine-Westphalia: Brilon-Hängeberg	Albach 1943	OLD08470	PZ959342/ PZ986713	
V. sublobata *	2323	Germany, Rhineland-Palatia: Idar-Oberstein	Albach 1503	OLD05784	PZ959347/ PZ986719	4x
*V. sublobata*	2321	Germany, Rhineland-Palatia: Mainz, University Campus	Albach 1508	OLD05789	PZ959346/ PZ986717	4x
*V. sublobata*	134	UK, Kew Gardens Road	Albach 116	K	AF509803/ AF486366	
*V. triloba*	2328	Austria, Wien, 10. district	Barta, T. s.n.	OLD00616	PZ959350/ PZ986724	
*V. triloba*	2331	Austria, Wien, 11. district	Barta, T., 2946	OLD01104	PZ959353/ PZ986727	
*V. triloba*	2332	Czech Republic : Strakonice: Rohozná: N of an agricultural road at the N edge of the Chlum Hill, NW of the village	Kúr, P., 87 − 16	OLD09945	PZ959354/ PZ986728	2x
*V. triloba .*	2337	Czech Republic, Distr. Klatovy, Horažďovice: S slope of the Na Hrádku hill	Kúr, P, 049/16 − 11	OLD09942	PZ959359/ PZ986733	3x
*V. triloba* (was *V. sublobata*)	2326	Czech Republic: Strakonice: Rohozná: N of an agricultural road at the N edge of the Chlum Hill, NW of the village	Kúr, P, 87 − 25	OLD09943	-/ PZ986722	2x
*V. triloba*	2333	Czech Republic: Strakonice: Rohozná: N of an agricultural road at the N edge of the Chlum Hill, NW of the village	Kúr, P., 86 − 27	OLD09944	PZ959355/ PZ986729	2x
*V. triloba*	2334	Greece, Lefkada	Fischer, 7.4.1999	WU	PZ959356/ PZ986730	
*V. triloba*	2329	Türkiye, Antalya: 3 km south of Korkuteli, among Thorny shrubbery	Studentexcursion, Inst. F. Spez. Bot., Joh.-Gutenberg Universität Mainz, D2-1	OLD00624	PZ959351/ PZ986725	
*V. triloba*	2330	Türkiye, Antalya: Termessos	Albach, D.C. & Köröklü, T., D6-3	OLD00625	PZ959352/ PZ986726	
*V. triloba*	2335	Türkiye, Aphrodisias	Albach 242	WU	AF509804/ AF513333	
*V. hederifolia x V. triloba*	2327	Czech Republic: Strakonice: Rohozná: N of an agricultural road at the N edge of the Chlum Hill, NW of the village	Kúr, P, 87 − 43	OLD09946	-/ PZ986723	4x
*V. crista-galli*	237	Georgia	Dolmkanov, 17.4.1983	TBS	AF509799/ AF486367	
*V. panormitana*	331	Greece, Lefkada	Fischer, 9.4.1999	WU	AF509800 AF513330	
*V. trichadena*	837	Türkiye	Mugla Sauer, 28.3.1983	WU	AY850093 AY850067	

**Table 3:** Information on specimens used only in the morphological analysis of herbarium specimens, including information on ploidy, FCM – flow cytometry

Taxon	Origin	Collector and collection number	Herbarium and herbarium number (if available)	Number of specimens analyzed	Ploidy estimated by FCM
*V. hederifolia*	Germany: Lower Saxony, Conneforde	Dirk Albach 1507	OLD 05961	9	6x
*V. hederifolia*	Czech Republic: Mnisek pod Brdy	Dirk Albach 1224	OLD 00550	4	6x
*V. hederifolia*	Germany: Hessia, Oberursel, Stierstädter Heide	Rüdiger Wittig 8938	OLD 05798	4	6x
*V. hederifolia*	Germany, Saxony, Dresden	Dirk Albach 1326	OLD 00551	2	6x
*V. hederifolia*	Germany: Lower Saxony, Conneforde	Dirk Albach 1507	OLD 05763	3	6x
*V. sublobata*	Germany: Northrhine-Westphalia, Assinghausen	Dirk Albach 1942	OLD 08471	3	4x
*V. sublobata*	Germany: Rhineland-Palatia, Idar Oberstein	Dirk Albach 1503	OLD 05784	2	4x
*V. sublobata*	Germany: Rhineland-Palatia, Mainz	Dirk Albach 1042	OLD 00357	4	4x
*V. sublobata*	Switzerland: Kanton Bern, Bern	Muriel Bendel	OLD 08472	3	4x
*V. sublobata*	Switzerland: Kanton Bern, Bern	Muriel Bendel	OLD 08473	5	4x
*V. sublobata*	Austria: Salzburg, Tennengau Hallein	Dirk Albach S857	OLD 05846	6	4x
*V. hederifolia*	Türkiye, Antalya	Albach, D.C. & Köröklü,T., D6-2	OLD00546	6	?
*V. hederifolia*	Türkiye, Antalya	Studentenexkursion, Inst. Spez. Bot., Joh.-Gutenberg Universität Mainz, D2-7a	OLD00547	9	?

The data set for DNA sequencing originated from 36 plants from different populations, partly from herbarium specimens and partly from silica-gel dried material collected in the field (Table 2). We sampled all known accepted species in the subsection and tried to include the geographic variation of the widespread species. We also checked GenBank for additional sequence data. While doing so, we noted that the sequence AJ548981 is a misidentified *Veronica arvensis*. Altogether, 36 plants were sampled, and the dataset was supplemented by previously published sequences (Table 2). Of these, among others, 13 individuals represent *V*. *hederifolia*, seven represent *V*. *sublobata*, and ten represent *V*. *triloba*. No new sequences of the rare *V. sibthorpioides* and *V. stewartii* could be generated in addition to those previously published [5]. The outgroup consisted of other members of *V*. subg. *Cochlidiosperma* (*V*. *panormitana*, *V*. *trichadena*, *V*. *crista-galli*). Nine of the specimens were also analyzed flow-cytometrically (Table 2).

The data set for flow cytometry, consists of three subsets: (a) 62 plants for the growth chamber experiments (67% of the surviving plants), (b) 66 individuals from 39 populations sampled throughout the distribution area of the species (Suppl. Table 2), and (c) a total of 145 plants were collected from 49 populations, 18 of them in multiple years, between 2016 and 2019 within the Czech Republic (Suppl. Table 3).

The dataset for the comparative growth chamber experiment consisted of 120 plants from one population. Plants were collected as young seedlings (one pair of leaves after the cotyledon) differentiated into two groups based on leaf color and thickness on the Oldenburg university grounds (53.1513°N, 8.1634°E) on February 14, 2024.

The dataset for the morphometry of plants from Czechia consisted of a total of 289 plants from 8 populations of *V*. *hederifolia*, 11 populations of *V*. *sublobata*, and 8 populations of *V*. *triloba* (3–15 plants per population).

The dataset for the morphometry of plants from Türkiye consisted of 60 plants from 13 populations (Suppl. Table 4). Of these, 22 specimens from five populations belonged to European *V. hederifolia*, 23 specimens from six populations belonged to *V. sublobata*, and 15 specimens from two Turkish populations identified as *V. hederifolia*. Apart from the latter, an additional six were analyzed phylogenetically using DNA sequences (Table 2).

### DNA sequence analysis

We extracted DNA from silica gel dried plants or herbarium specimens crushed in a MM200 mill (Retsch GmbH, Haan, Germany) using the innuPREP plant DNA kit (Analytik Jena GmbH, Jena, Germany) following the manufacturer’s protocol with 20 mg of dry plant material. PCR of the nuclear ribosomal ITS region (including parts of the 18S gene, ITS1, 5.8S, and ITS2) and plastid *trn*L-F region (including the *trn*L intron, the 3’ exon of the *trn*L gene, the *trn*L-F spacer and parts of the *trn*F gene) was conducted using primers ITS4 [31] and ITSA [32] and c and f [33], respectively. The PCR mix contained 1 µl DNA, 1 µl of each primer at 10pmol/µl, 0.2 µl myTaq (5U/µl; Bioline, Memphis, TN, USA), 4 µl 5x myTaq buffer, 1 µl bovine serum albumin and 16.8 µl ddH_2_O. PCRs were conducted on a T-Professional standard thermocycler (Analytik Jena GmbH, Jena, Germany) with 35 cycles of 20 s at 94 °C, followed by 40 s at 51 °C and 20 s at 72 °C. At the end, reactions were kept at 72 °C for five minutes before storing them at 4 °C. PCR products were sent for Sanger sequencing to LGC Genomics (Berlin, Germany).

DNA sequences were checked and aligned in Geneious v. 8.17 using the default options of MAFFT. New sequences were combined with previously published sequences. Sequences were phylogenetically investigated in PAUP v. 4.0a168 [34] using maximum parsimony. Heuristic searches were conducted using TBR branch swapping and limited to 100 optimal trees per search. Subsequently, 1000 bootstrap replicates (maximum 1000 trees per replicate) were analyzed to investigate branch support. A maximum likelihood analysis with 1000 ultrafast bootstrap replicates and a GTR + Γ-model was run using W-IQ-TREE [35] on https://www.hiv.lanl.gov. A Bayesian analysis using MrBayes was run twice with two chains of 1 million generations, sampling every 5000th generation and a GTR + Γ-model on the Galaxy server [36].

### Genome size estimation

The genome size of all plants collected was analyzed using flow cytometry. Only fresh tissues were used. The simplified two-step procedure of nuclear isolation and staining [37] modified for plant tissues [38] was employed. Fresh leaves together with an appropriate amount of the internal standard were chopped with a razor blade in a Petri dish containing 0.4/1.1 ml (Czech recipe/German recipe) of ice-cold Otto I buffer (0.1 M citric acid, 0.5% Tween 20). *Bellis perennis* (2 C = 3.38 pg) [39] was used as an internal standard for the plants from Czechia, *Solanum pseudocapsicum* (2 C = 2.59 pg) [40], *Zea mays* CE-777 (2 C = 5.43 pg, for all specimens of the comparative treatment [41]), , *Pisum sativum* (2 C = 9.09 pg; [42]) or *Hedychium gardnerianum* (2 C = 4.02 pg; [43]) for the other plants. The suspension was filtered through a 42 μm/30µm nylon mesh and after a five-minute incubation at room temperature or 30 min at 37 °C, 0.8/2 ml of staining solution containing Otto II buffer (0.4 M Na_2_HPO_4_ · 12 H_2_O), fluorochrome propidium-iodide (PI; 50 µg/ml), β-mercaptoethanol (2 µl/ml), and RNase (50 µg/ml) were added to the whole amount/half the amount. Up to five plants were pooled per sample, with additional samples analyzed individually when required. Samples were run through a Partec CyFlow ML/SL flow cytometer (Sysmex, Münster, Germany) equipped with a 532 nm (green) diode-pumped solid-state laser (100 mW output) as the excitation source. The fluorescence intensity of 3500–5000 particles was recorded. The fluorescence histograms were evaluated using the flowPloidy R package [44] and FloMax 2.52 (Sysmex, Münster, Germany), with mean fluorescence and coefficient of variation recorded for all fluorescent peaks. Only analyses with coefficients of variation below 5% were considered for genome size estimates. For each sample in which the coefficient of variation (CV) of the sample or standard peaks was below 5%, the monoploid (1Cx) genome size was calculated. In addition, we decided to test the hypothesis that *V*. *hederifolia* is an allohexaploid hybrid of *V*. *triloba* and *V*. *sublobata* by modelling the genome size of a hypothetical allohexaploid by combining the full somatic genome sizes of all diploid–tetraploid pairs in the dataset, thereby emulating unreduced gametes, and included these values in subsequent comparisons. Differences in the 1Cx genome size among groups were tested using the Kruskal–Wallis ANOVA, followed by pairwise Wilcoxon tests, implemented in R 4.4.3 [45].

### Comparative growth chamber experiment and phenotypic characterization

For stabilization and root formation, the pricked-out seedlings for the growth chamber experiment were kept for one week in a greenhouse (3 l perlite to 70 l growing medium, pH 5.5, N 140 mg/l, KCl 1.0 g/l, P_2_O_5_ 100 mg/l) and were subsequently placed in a climate chamber (polyklima Climatic Grow System, polyklima, Freising, Germany) at 21 °C and 60% humidity (70% from day 6 onwards). The lighting in the climate chamber was provided by white LED panels for plant cultivation (16 h light/day).

Five treatments with 24 plants each were set up in the chamber, which differed either in their light intensity or their water availability. Experiment A (shade) was set to 22.7 µmol×m^− 2^×s^− 1^, measured using a Fieldscout Quantum / Foot-Candle-Meter (Spectrum Technologies, Aurora, IL, USA). Treatments B, C and D were each exposed to a medium light intensity (80.0–82.6 µmol×m^− 2^×s^− 1^), and treatment E (bright) was exposed to 185 µmol×m^− 2^×s^− 1^. All intensities were measured at the top of the plant at the beginning. Moisture content (volume percent) was measured (Moisture Meter type HH2, Delta-T Devices, Cambridge, Great Britain) three days a week. A fixed irrigation schedule was planned in which the “dry” treatment (B) was kept at 16% soil moisture, at which point plants showed wilted leaves but would not die. The “moist” treatments A, C, E received about double the amount of water (soil moisture 30–50%, max 12% difference between treatments), whereas the pots of the wet treatment (D) stayed in water-filled trays, constantly refilled to a water level of 2–3 cm (70% soil moisture).

Of 120 plants, 92 survived and were assigned to two groups based on dark green, thick leaves with long trichomes (defining the species *V. hederifolia*) and light green, thin leaves with short trichomes (defining the species *V. sublobata*) following Fischer [6] (Table 4). Leaf samples were taken as soon as fruits formed (March 18–22, 2024). In treatment A, only small flower buds developed but were sampled in parallel with others.

**Table 4:** Surviving plants of the five treatments. In brackets the number of plants used in flow cytometry

Treatment	Light green	Dark green	Total
A (dark)	13 (6)	6 (6)	19 (12)
B (dry)	12 (8)	6 (6)	18 (14)
C (medium)	15 (7)	3 (3)	18 (10)
D (wet)	12 (8)	5 (5)	17 (13)
E (bright)	13 (7)	6 (6)	19 (13)
Total	65 (36)	27 (27)	91 (62)

Four leaves were analyzed from each of the plants: the uppermost fully developed leaf (top), two leaves from the center (center 1, 2) and one of the lowest opposite leaves (bottom). All four leaves of the plant were scanned with a digital scanner (CanoScan LiDE 400, Canon, Tokyo, Japan) at 600 dpi resolution together with a color chart, a ruler with millimeter graduations and a 30 mm × 30 mm dark square. All characters were determined with ImageJ (Schneider et al. 2012) on white-balanced images.

Particle analysis was used to determine the leaf area of the individual leaves in mm^2^ based on black-and-white images. Dried leaves were weighed in mg by a precision balance (QUINTIX 125D-1 S, Satorius, Göttingen, Germany) and the specific leaf area (SLA) was measured as the ratio of both. Trichome density (TD) and the trichome length (TL) were measured on two leaves from the middle section of the shoot axis. These were photographed under a binocular microscope (SZX16, Olympus, Tokyo, Japan) with an accompanying camera (UC 30, Olympus, Tokyo, Japan) at 8x magnification (cellSens program, version 2.2, Olympus, Tokyo, Japan). All hairs were counted on a 3 mm × 3 mm area towards the leaf tip, and at least five hairs were measured in length. The shape of the middle lobe was determined by the ratio of width to length of the lobe [6]. The width of the lobe was based on the straight line between the two lowest incision points of the uppermost left and notch to the right of the lobe. The lobe length was defined as the longest line perpendicular to the previously drawn width line. In order to determine the depth of the notch and its degree, the four scanned leaves (leaf position top, middle 1, middle 2, bottom) were measured. For each leaf, the depth of the deepest notch of the blade and the length of the leaf blade (without petiole) were measured with the program ImageJ [46]. The measurements were performed using the ROI-Manager of ImageJ and the results were saved in an Excel file. To determine the number of lobes per leaf, the number of incisions of a leaf was counted. To determine the RGB values of the adaxial side of the leaf, values were determined with white-balance treated scans of the four leaves. Color was determined in a rectangle measuring 2.5 mm × 1.5 mm positioned next to the mid-vein as far to the top as possible. The values of the areas were entered in the ROI Manager and then the ImageJ plug-in “RGB-Measure” [46] was used to calculate the mean values for red, green and blue in the selected area. Elliptic Fourier analysis was used to analyze the shape of the scanned leaves using the program Shape (Iwata and Ukai 2002) in the blue color channel and without a square reference scale. A description of the leaf shape (Fourier descriptors = EFD) was created via chain codes and these were used in a principal component analysis (PCA). Plants were additionally, if available, investigated for the pedicel/calyx length ratio in fruit and the hairiness of the pedicel. If open flowers were present, the flower color was also noted.

### Morphometric analysis of plants from Czechia

Multivariate morphometric analyses were conducted to investigate patterns of morphological variation among the studied representatives of the *V. hederifolia*-group. Two sets of morphological characters were analyzed.

The first set comprised classical morphological characters selected on the basis of our observations, identification keys, floras, and previous taxonomic studies. This set included 17 quantitative characters and one binary character. For characters allowing repeated measurements (i.e., characters occurring multiple times on an individual plant), three measurements were taken per individual, and their arithmetic mean was used as the representative value in all morphometric analyses. The length of hairs on the calyx was measured five times per flower. All characters were measured from high-resolution scans using ImageJ software [46].

All characters, except pedicel length and pedicel hair arrangement (which were left untransformed), were subjected to the Yeo–Johnson transformation to improve normality prior to analyses assuming normally distributed variables (PCA, LDA, and Pearson correlations). Pearson correlation coefficients were calculated for all pairs of characters to identify strongly correlated variables; from each pair with |r| > 0.95, only one character was retained for subsequent analyses (i.e., pedicel length / calyx length ratio and pedicel length (*r* = 0.954). Principal component analysis (PCA) was then applied to explore overall patterns of morphological variation among individuals. Prior to PCA, all variables were standardized to zero mean and unit variance.

Linear discriminant analysis (LDA), which derives linear combinations of variables that maximize separation among predefined groups, was performed to assess the discriminatory power of morphometric characters for distinguishing groups defined by the flow cytometric results. Forward selection of characters was conducted using non-parametric Monte Carlo permutation tests (999 permutations) to identify the most informative character combinations. Predictive performance was evaluated using cross-validated classificatory LDA with leave-one-population-out cross-validation.

The predictive power of morphological characters was further assessed using classification and regression trees (CART) and the JRip algorithm. CART provides a non-parametric alternative to discriminant analysis by constructing a hierarchical classification based on successive univariate splits, visualized as an easily interpretable tree. Although still relatively uncommon in plant taxonomy, this approach has proven effective in several taxonomic studies (e.g [47–50]). JRip (RIPPER) rule sets represent an alternative to both discriminant analysis and classification trees by inducing a set of propositional if–then rules through incremental growing and pruning to minimize classification error, yielding an interpretable rule-based model rather than a hierarchical tree structure [51]. To our knowledge, this method has not yet been applied in plant taxonomy. Predictive performance of all the methods was evaluated using leave-one-population-out cross-validation.

The second set of characters described flower color. One flower per individual was photographed in situ using a photographic grey card (neutral grey, RGB = 128, 128, 128) as the background for color calibration. The white balance of each photograph was calibrated using the grey card, and the color of a representative part of the flower (equally illuminated as the grey card and not shaded) was extracted. Flower color was quantified in a perception-aware manner as follows: images were converted to the HSV color space, and greenish hues caused by contamination from surrounding tissues were removed by retaining only pixels with hue ≥ 100 (OpenCV HSV scale). For the remaining pixels, a salience score was calculated as the product of saturation and value (S × V). Pixels were ranked in descending order by this score, the top 5% were selected, and the mean HSV values of this subset were used as the representative color. Flower color was thus expressed as a triplet of H, S, and V values. Based on our observations, this approach captured perceived flower color more faithfully than simple color averaging.

Linear discriminant analysis was again performed to assess the discriminatory power of the color components in distinguishing groups defined by the flow cytometric results. No forward selection of characters was conducted, as the HSV color components function as an integrated set. Instead, a non-parametric Monte Carlo permutation test (999 permutations) was used to assess the significance of the overall model. Predictive performance was evaluated using cross-validated classificatory LDA with leave-one-population-out cross-validation.

PCA, LDA, classificatory discriminant analysis, and Pearson correlation coefficients were computed using MorphoTools 1.1 [52]. Classification trees were constructed using the rpart package [53] in R 4.4.3 [45], with the minimum split parameter (minsplit) set to 1 and the initial complexity parameter (cp.) set to 0.001; the final tree was selected using the 1-SE rule [54]. JRip was calculated using the RWeka package [55] with default parameter settings.

In addition to the classical morphometric analyses, differences in leaf shape were assessed using an outline analysis. Leaf shape variation was quantified using elliptic Fourier approximation [56] as implemented in the R package Momocs [57]. Elliptic Fourier descriptors (EFDs) were centered and scaled to zero mean and unit variance. Principal component (PC) scores were calculated from the standardized EFDs to visualize patterns of leaf shape variation.

These PC scores were subsequently used in linear discriminant analysis (LDA) and classificatory discriminant analysis (CDA) in MorphoTools 1.1 [52] to test for interspecific differences in leaf shape. Model significance was assessed using a permutation test with 999 permutations.

### Morphometry of Turkish *Veronica hederifolia*

Based on the deviant position of Turkish *V. hederifolia* (see below), the Turkish specimens were included in a morphological analysis to identify characters that may distinguish them from European populations of *V. hederifolia*. Since some characters analyzed in the growth chamber experiment could not be estimated in herbarium specimens, we restricted the analysis to ten characters and two ratios: leaf length and width, length and width of the central lobe of the leaf and their ratio, leaf incision depth, thickness of the basal hair on the calyx, pedicel length and calyx length and their ratio, and flower length and width.

Herbarium specimens were investigated and measured using an SZX16 research stereo zoom microscope (Olympus). Images were recorded using a Digital Color Camera UC30 (Olympus). The samples were measured using either “cellSens Dimension” (Olympus) or “ImageJ” (Wayne Rasband). All statistical analyses were carried out in R [45] with the packages ggplot2 [58], tidyverse [59], psych [60], gridExtra [61], and patchwork [62]. The datasets were first tested for normality using the Shapiro–Wilk test. Normally distributed data were tested for significant differences using two-sample t-tests for pairwise comparisons among European *V. hederifolia*, *V. sublobata*, and Turkish *V. hederifolia* s.l., and ANOVA for simultaneous comparisons of all three taxa. Non-normally distributed data were tested using Mann–Whitney U tests for pairwise comparisons among European *V. hederifolia*, *V. sublobata*, and Turkish *V. hederifolia* s.l., and Kruskal–Wallis tests for simultaneous comparisons of all three taxa. Measurements were visualized in a biplot combining the scores of the principal component analysis of the morphological measurements with the loadings.

## Results

### DNA sequence analysis

**Fig. 1:**
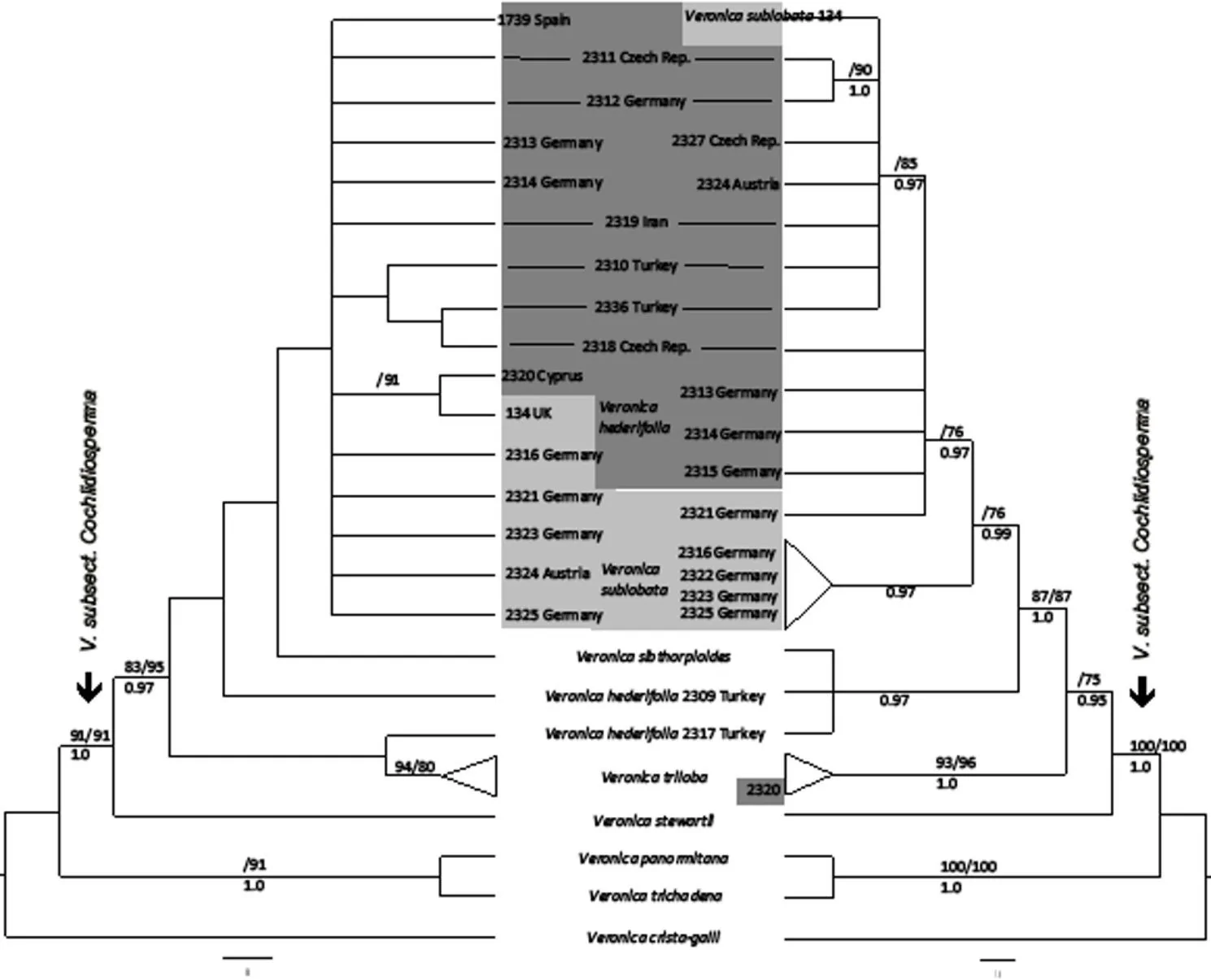
Most parsimonious trees from the phylogenetic analyses of the nrITS region (left) and plastid *trn*L-F region (right). Samples were assigned to species based on morphology. The light grey box highlights samples identified as *V. sublobata*, and the dark grey box highlights those identified as *V. hederifolia* s.str. Support values above the branches indicate parsimony/likelihood bootstrap support (if above 70%), those below indicate Bayesian posterior probabilities (if above 0.9)

The nrDNA dataset had 686 characters, of which 64 were parsimony-informative. The 500 optimal trees had 251 steps (consistency index (CI) = 0.76, retention index (RI) = 0.83). The *trn*L-F dataset had 1074 characters, of which 106 were parsimony-informative. The 180 optimal trees had 321 steps (CI = 0.87, RI = 0.90). The phylogenetic analyses of nuclear ribosomal (nrDNA) and plastid DNA (cpDNA) retrieved a monophyletic *V*. subsect. *Cochlidiosperma* with *V. stewartii* branching first (Fig. 1). The central European samples overwhelmingly fell into two clades (Fig. 1) corresponding to *V*. *triloba*, and a combined clade of *V*. *sublobata* and *V*. *hederifolia* s.str. The fifth accepted species, *V. sibthorpioides*, is sister to the latter clade (Fig. 1). A number of samples fell outside these clades (Fig. 1), most notably samples 2309 and 2317 collected near Antalya in southern Türkiye. In the nrITS analysis, sample 2317 was sister to *V. triloba* using parsimony but sister to the rest using likelihood, whereas sample 2309 was sister to the clade comprising *V. sibthorpioides*, *V. sublobata*, and *V. hederifolia* using parsimony and sister to V. sibthorpioides using likelihood. The relationships of these two samples did not receive strong support (BS < 70%, PP < 0.9). In the cpDNA analysis, samples 2309 and 2317 formed a clade together with *V. sibthorpioides*.

### Genome size estimation

Most of the specimens analyzed could clearly be assigned to three different size classes, which we inferred to be the diploid, tetraploid, and hexaploid level. In the mixed-cytotype population from Oldenburg used in the growth chamber experiment, the 2 C values of 26 individuals ranged between 4.26 pg and 4.40 pg, and those of 36 individuals between 2.92 pg and 3.04 pg. Based on previously published data, we assigned these measurements the hexaploid and tetraploid levels, respectively. Among the wider sampling, variation between the two groups increased to 2.82–3.21 pg for the tetraploids and 4.02–4.40 pg for the hexaploids (Suppl. Table 2; Suppl. Figures 1, 2). Among the Czech populations, we measured 214 individuals, 73 corresponding to the diploid level, 68 to the tetraploid level, and 72 to the hexaploid level with several mixed-cytotype populations (Suppl. Tables 3, 5). A single triploid plant was found (1.88 pg, Suppl. Tables 3, 5).

**Fig. 2:**
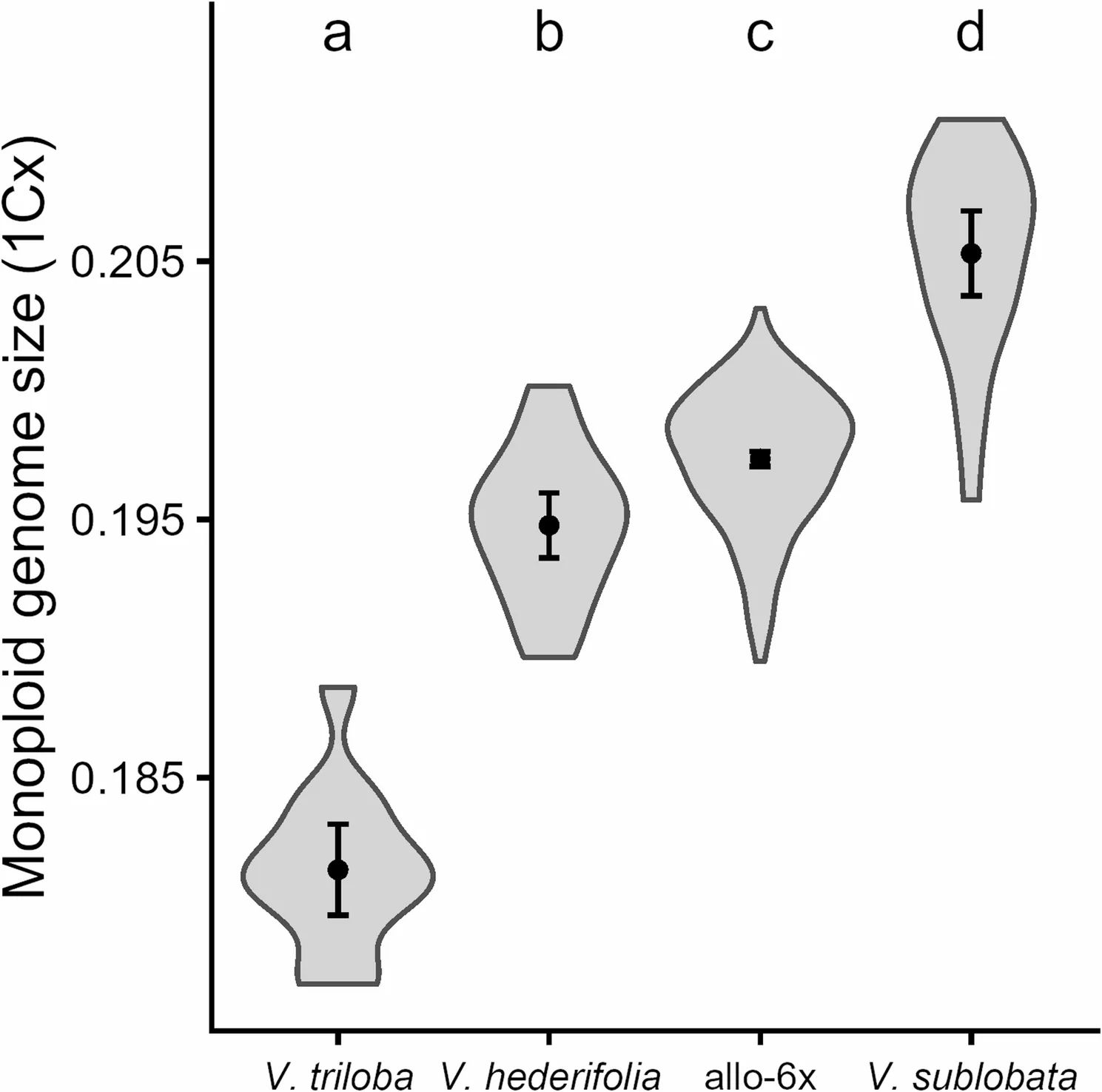
Violin plot showing the monoploid genome sizes of the analyzed species in the *Veronica hederifolia* group. The central dot represents the mean, and the whiskers indicate the 95% confidence interval of the mean. Letters above each group denote statistically significant differences in genome size among groups based on pairwise Wilcoxon tests

The 1Cx genome sizes differed significantly among all groups, including the simulated hypothetical allohexaploids (Kruskal–Wallis ANOVA: χ² = 101.07, df = 3, *p* < 0.001). Specifically, hexaploids differed significantly from the hypothetical allohexaploids (Wilcoxon test: *p* < 0.001), exhibiting, on average, a 1.02% smaller genome size (Fig. 2).

### Morphological analysis of growth chamber experiment

**Fig. 3:**
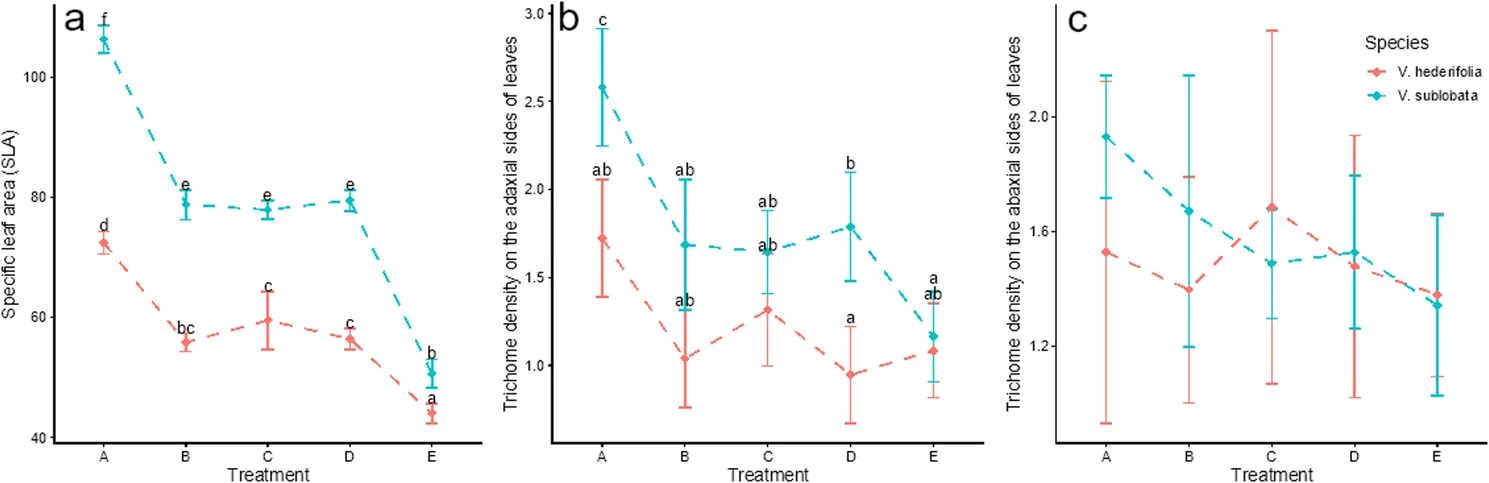
**a** Specific leaf area (SLA), **b** trichome density on the adaxial leaf surface, and **c** trichome density on the abaxial leaf surface across treatments for each species in the growth chamber experiment. Points show group means, dashed lines connect treatment means within species, and error bars represent 95% confidence intervals calculated from the raw data. Letters above points indicate Tukey-adjusted compact letter display groups based on estimated marginal means from the Treatment × Species ANOVA; groups sharing a letter are not significantly different at α = 0.05

*Veronica hederifolia* had significantly lower SLA values than *V*. *sublobata* in all treatments (*p* < 0.001; Table 5). In both, *V*. *sublobata* and *V*. *hederifolia*, the mean of the specific leaf area (SLA) of treatment A (dark) was the highest (Suppl. Tables 6, 7), and that of treatment E (light) the lowest (Fig. 3; Suppl. Table 6; *p* < 0.001).

**Table 5:** Results of two-way ANOVA testing the effects of treatment, species, and their interaction on specific leaf area (SLA) and trichome density on the adaxial and abaxial leaf surfaces

Response variable	Source of variation	df	F	*p*
Specific leaf area (SLA)	Treatment	4	453.95	< 0.001
	Species	1	697.83	< 0.001
	Treatment × Species	4	33.91	< 0.001
Trichome density — adaxial	Treatment	4	15.43	< 0.001
	Species	1	25.38	< 0.001
	Treatment × Species	4	1.94	0.106
Trichome density — abaxial	Treatment	4	1.92	0.109
	Species	1	1.10	0.296
	Treatment × Species	4	0.76	0.551

Across treatments, adaxial TD of *V*. *sublobata* (x̃ = 1.56) was significantly higher (*p* < 0.001, Suppl. Table 6) than that of *V*. *hederifolia* (x̃ = 1.11). In contrast, abaxial TD did not differ significantly between species (*V*. *hederifolia*: x̃ = 1.33, σ = 0.66; *V*. *sublobata*: x̃ = 1.44, σ = 0.75, Suppl. Table 6). Both species exhibited significantly higher trichome density (TD) on the adaxial leaf surface under treatment A (shade) than under treatment E (bright light) (*p* < 0.001; Fig. 3b, c, Suppl. Tables 6, 7). Comparisons of treatment effects between species were not significant on either leaf surface (Table 5).

Between species, the adaxial trichomes of *V*. *hederifolia* were significantly longer in all treatments (*p* < 0.001) than those of *V*. *sublobata* (Suppl. Figure 3, Suppl. Tables 6, 7). The difference was also visible abaxially, but it was statistically significant only for the light-influenced treatments A and E (A: *p* < 0.038; E: *p* < 0.0032; Suppl. Table 7).

The shape of the central lobe was described by the ratio of length to width. It did not show a difference between treatments and species (Suppl. Tables 8, 9). There was, however, a notable difference between leaf positions with the upper leaves having a notably elongated middle lobe gradually decreasing in the lower leaves (Suppl. Figure 4, Suppl. Table 8). The degree of incision (DI) did not differ between species and treatments (Suppl. Tables 8, 9), but leaf position played a prominent role, with the lower leaves being less deeply notched (Suppl. Table 10).

In *V*. *sublobata*, the number of lobes (3–8) was significantly higher across all treatments than that of *V*. *hederifolia* (2–7) (*p* < 0.001). The number of lobes did not differ between treatments in *V*. *sublobata*, but *V*. *hederifolia* had a significantly higher number in treatment E (light) compared with treatment B (dry) and treatment D (wet) (*p* = 0.012 in each case; Suppl. Tables 8, 9). The average leaf area of *V*. *sublobata* was above that of *V*. *hederifolia* in all five treatments. In order to check whether there is a correlation between leaf size and the number of lobes, a correlation analysis was carried out, which showed no such correlation either across species or within species (*p* > 0.05). In *V*. *hederifolia*, there was a clear decrease in lobe number from top to bottom, which was not as clear in *V*. *sublobata*, leading to a clear difference in lobe number between the two species at the bottom but not at the top (Suppl. Figure 5, Suppl. Table 10).

The two species differed significantly in all color channels—both within all treatments (*p* < 0.001) and across treatments (*p* < 0.001) (Suppl. Figure 6). For light-dependent treatments, red and green exhibited decreasing reflectance from low light availability (Treatment A) to high light intensity (Treatment E). For the water-dependent treatments, the values of green and red differed less but increased from treatment B (dry) to treatment D (wet; *V*. *hederifolia*, *p* = 0.003; *V*. *sublobata*, *p* < 0.001).

In the principal component analysis (PCA) carried out for the symmetrical components of the leaf shape, the main components PC1–PC4 covered around 93% of the shape variants (Suppl. Figures 7, 8), with the main share (70.55%) of the variation represented by PC1. This component expressed the shape of the lower half of the leaf in interaction with the petiole length: positive values indicate pronounced broad “winged” lateral lobes with a short petiole, negative values a stout, broadly ovoid blade with a flat central lobe and a long petiole. PC2 (13.3%) described differences in the depth of the middle lobe and the shape of the upper half of the ridge in relation to petiole length — positive values represent a round leaf shape with an elongated middle lobe and a short petiole; negative values represent a flat middle lobe with a long petiole. When PC1 was plotted against PC2 (84% of the variation), the significantly larger ellipse of *V*. *hederifolia* (95% of the data) contained the ellipse of *V*. *sublobata* completely (Suppl. Figure 8). At the treatment level (PC1 vs. PC2), there was a large overlap of the ellipses of the individual treatments. At the leaf position level, the ellipses of the leaf positions “at the top” differed from a cross-species perspective (Suppl. Figure 8). Upper leaves tend to have shorter petioles and a proportionally broader (“winged”) lower half of the blade; the lower leaves, on the other hand, have a rounder leaf shape with somewhat longer petioles. The species differed more with respect to PC4, which represents a deeper central lobe and a rounder transition from lateral to central lobe (Suppl. Figure 7).

For the character pedicel/calyx length ratio, the two species overlapped in terms of the expression of characteristics between 2.5 and 5.0. However, none of the *V*. *hederifolia* individuals showed a ratio of > 5.0 (Suppl. Figure 9). Only one *V*. *sublobata* specimen had a ratio of < 2.0. Trichomes outside the hairline of the fruit stalk were found in all specimens, but *V*. *hederifolia* specimens had noticeably fewer hairs than *V*. *sublobata*. Flowers of *V*. *hederifolia* specimens were predominantly violet-colored, and one quarter were white. In *V*. *sublobata*, flowers were predominantly white and sometimes light blue.

### Morphometric analysis of plants from Czechia

**Fig. 4:**
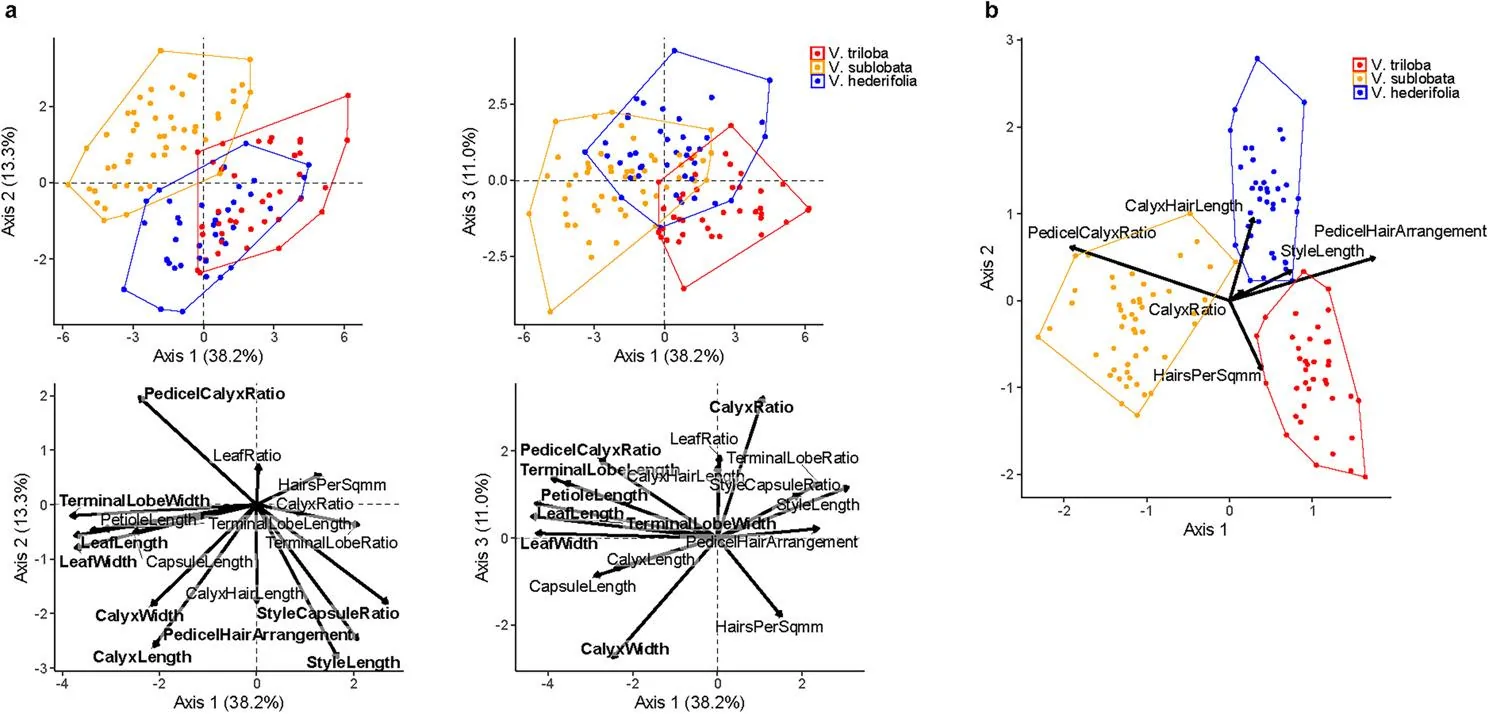
Results of multivariate morphometric analyses based on 18 morphological characters for species of the *Veronica hederifolia* group: **a** principal component analysis (PCA) and **b** linear discriminant analysis (LDA). For abbreviations of characters, see Suppl. Table 11. Numbers in parentheses denote the percentages of variation explained by each axis

The PCA showed a clear separation of *V*. *triloba*, *V*. *sublobata*, and *V*. *hederifolia* (Fig. 4a). This pattern was corroborated by linear discriminant analysis, which likewise showed a pronounced separation of the three species (Fig. 4b). Marginal effects of all except three characters (calyx length / width ratio, leaf length / width ratio, and calyx width) in the LDA were significant. Forward selection identified six characters that contributed most to the separation of the species, of which the pedicel length / calyx length ratio, length of calyx hairs, and pedicel hair arrangement topped the selection (Suppl. Table 11).

**Fig. 5:**
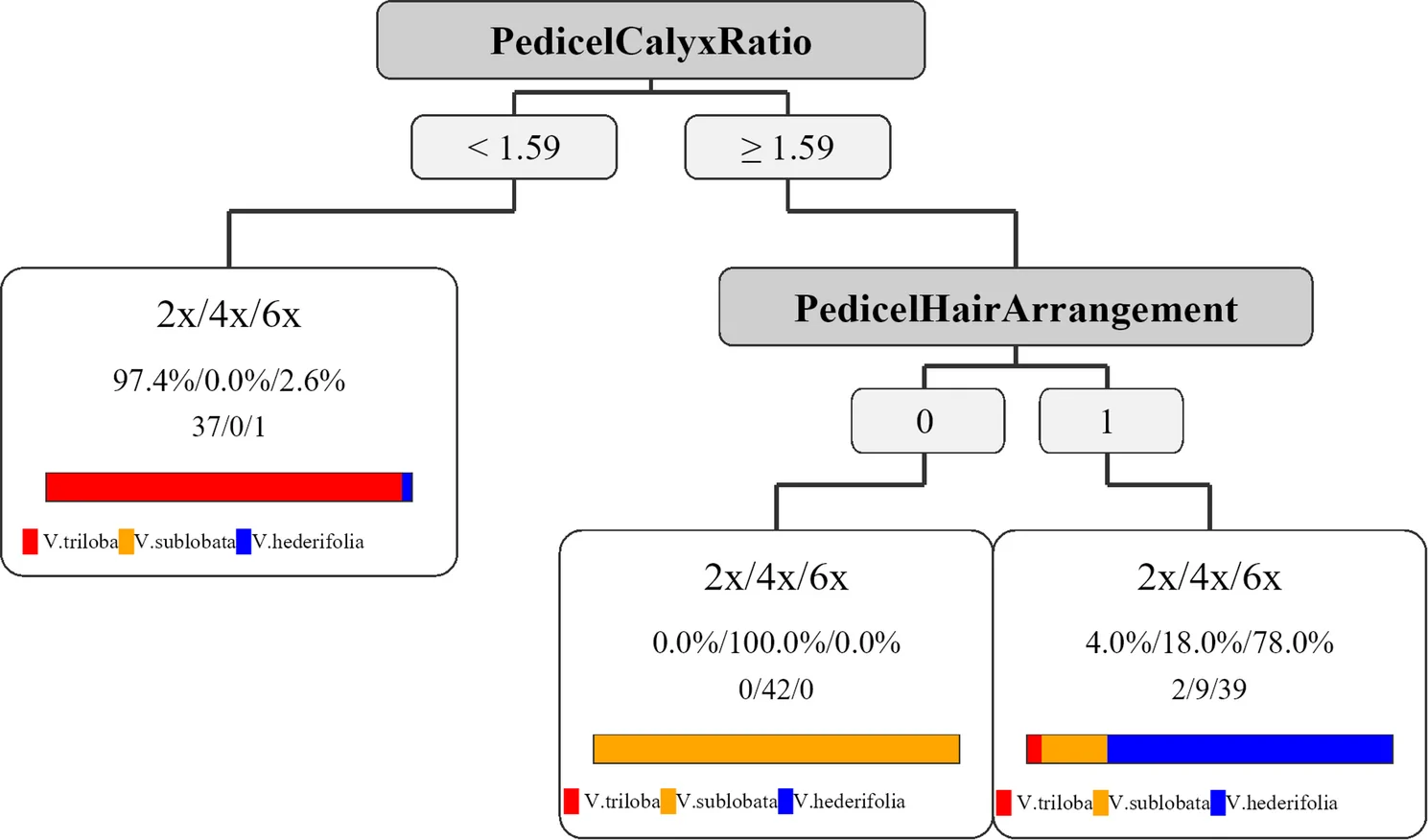
Classification tree of the species of the *V. hederifolia* group

The pedicel length / calyx length ratio and pedicel hair arrangement were also the characters selected by the classification trees (Fig. 5). The JRip method resulted in the following set of rules.

1a. Pedicel–calyx ratio ≤ 1.59 → *V*. *triloba*.

1b. Pedicel–calyx ratio > 1.59 → go to 2.

2a. Hairs on pedicels arranged in one row and calyx hair length ≥ 0.60 mm → *V*. *hederifolia*.

2b. Otherwise → *V*. *sublobata*.

Leave-one-population-out cross-validation showed the highest predictive power of the LDA with an accuracy of 95.38%, followed by JRip (accuracy of 92.31%), and classification trees (accuracy of 90.00%) (Suppl. Table 12).

The permutation test of the LDA model based on the flower color dataset was highly significant (*p* < 0.001). The species were clearly separated by the LDA (Suppl. Figure 10), although the predictive power of flower color was lower than that of the morphological characters, with an overall classification accuracy of 78.5% in the classificatory discriminant analysis (Suppl. Table 13). Although overlap in flower color among species was considerable, flower color most effectively separated *V*. *triloba* from *V*. *sublobata*. In particular, *V*. *triloba* exhibited the darkest and deepest blue flowers, whereas pinkish hues predominated in *V*. *sublobata*. *Veronica hederifolia*, in contrast, displayed intermediate bluish–greyish–pinkish flower colors (Suppl. Figure 10).

The first 13 harmonics, accounting for 99% of the total harmonic power, were retained to approximate leaf shape. Principal component analysis of the harmonic coefficients revealed substantial overlap among the species. Only a small subset of extreme morphotypes of *V*. *triloba*, characterized by clearly trilobed leaves, could be distinguished from the remaining species (Suppl. Figure 11). Linear discriminant analysis of the PC scores still indicated significant differentiation among species (*p* < 0.001); however, the overall classification accuracy of the classificatory discriminant analysis was low, reaching only 47.8% (Suppl. Table 14).

### Turkish *Veronica hederifolia*

**Fig. 6:**
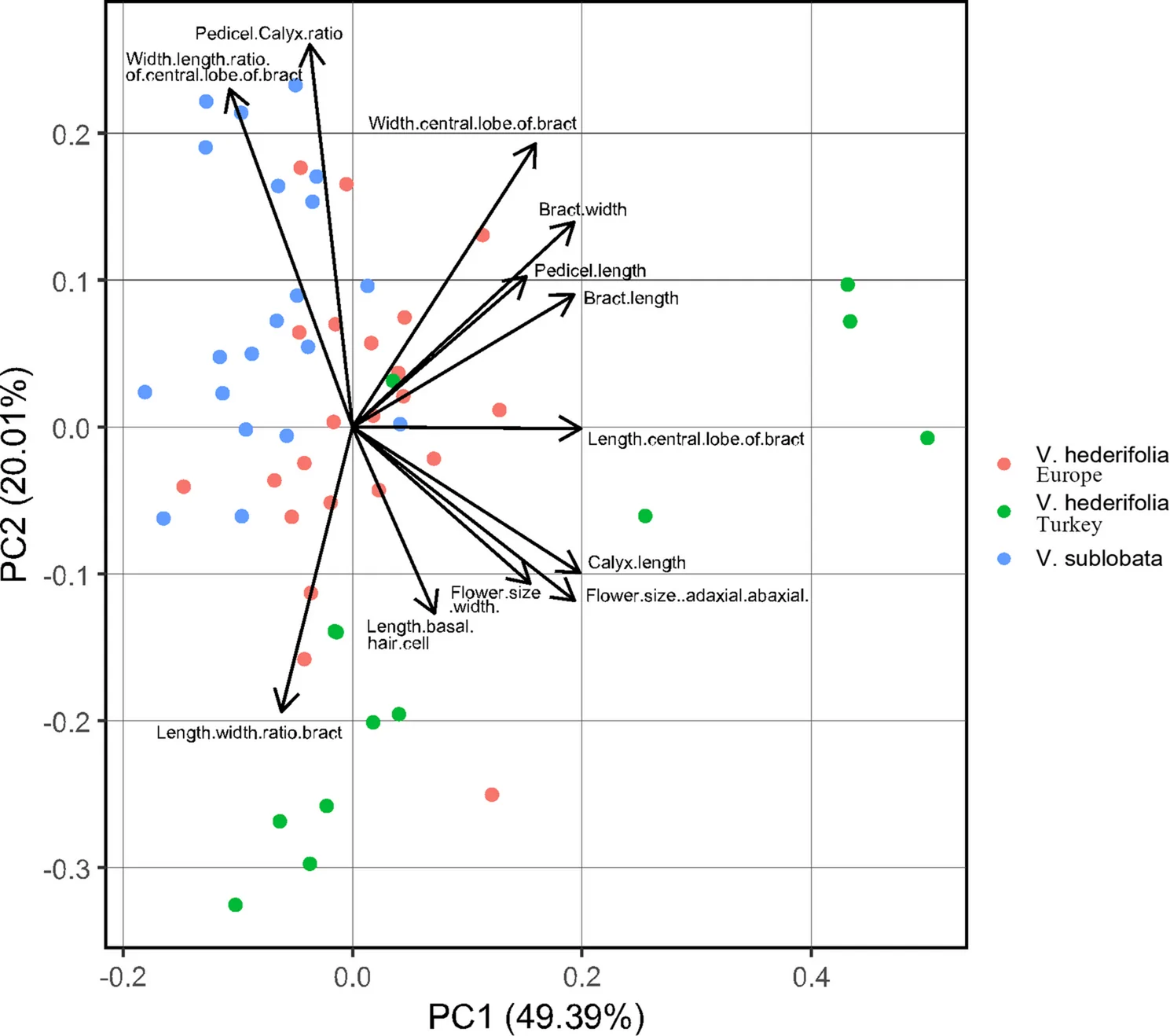
PCA biplot of the morphological measurements based on herbarium specimens for European *V. hederifolia*, Turkish *V. hederifolia* and *V. sublobata*

**Fig. 7:**
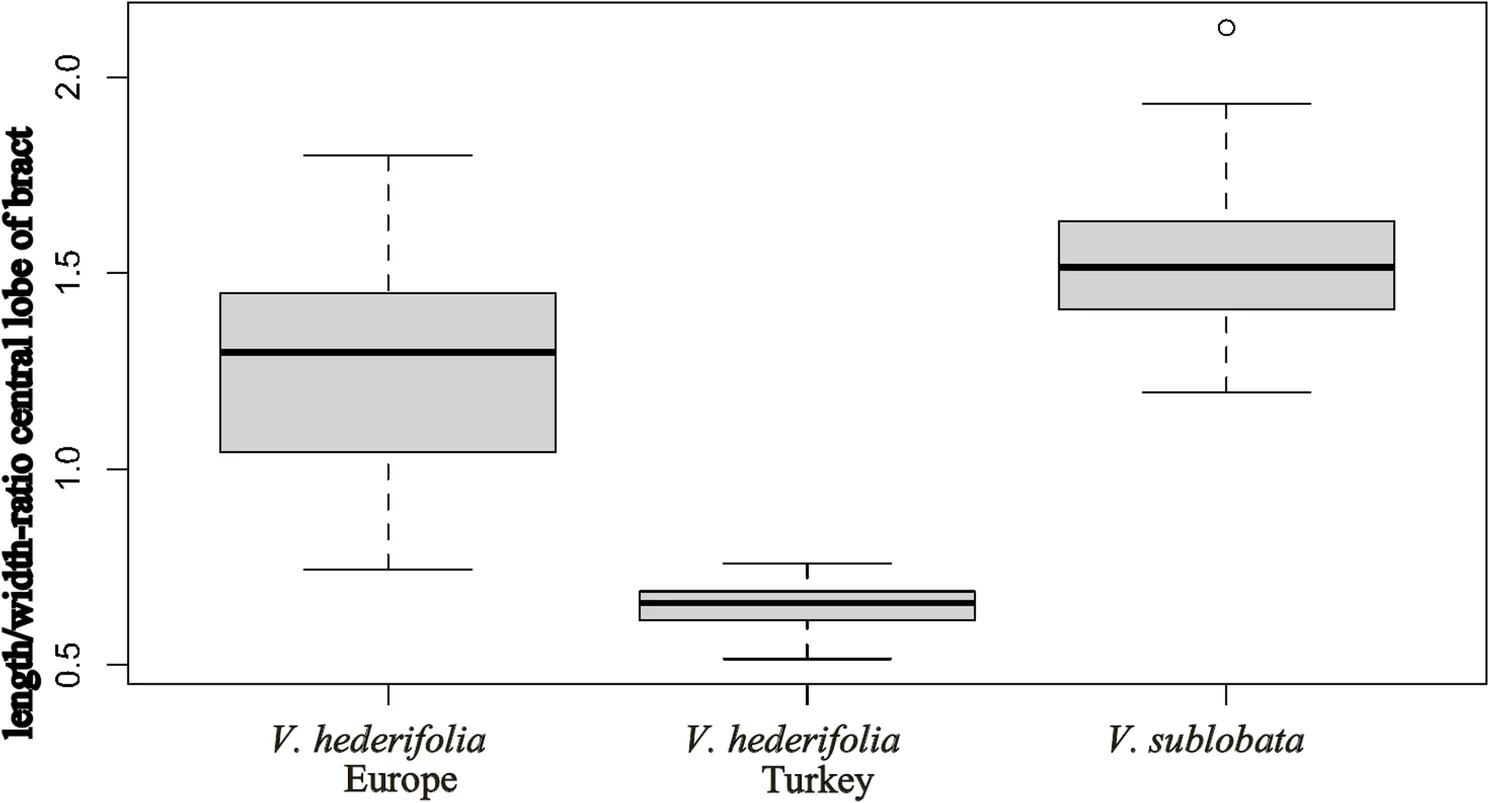
Box plot of length/width ratio of the central lobe of the leaves based on herbarium specimens for European* V.* hederifolia, Turkish* V. *hederifolia and* V. sublobata*

**Fig. 8:**
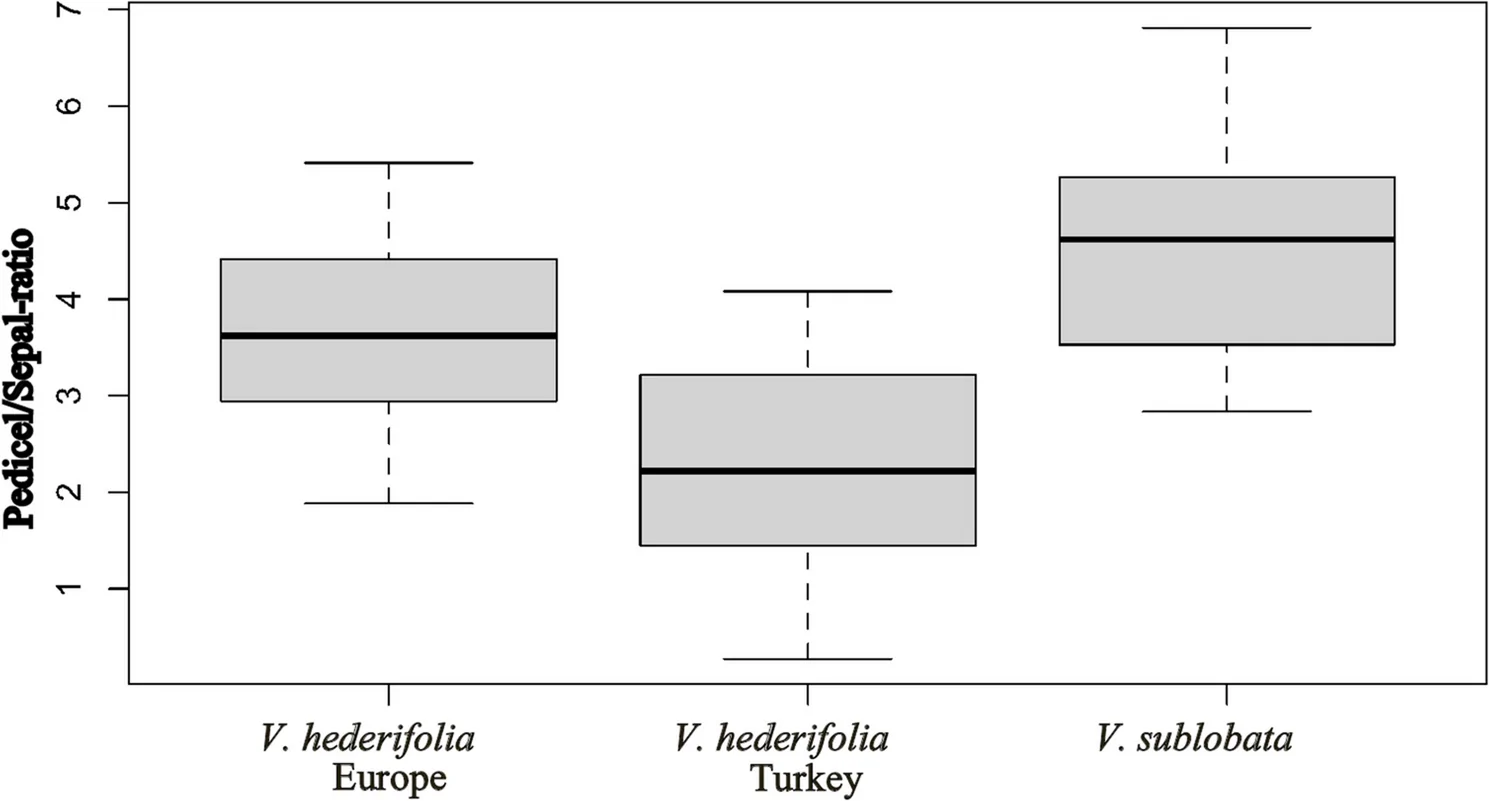
Box plot of pedicel/calyx length ratios based on herbarium specimens for European *V. hederifoli*a, Turkish *V. hederifolia* and *V. sublobata*.

In the morphological dataset including Turkish specimens, significant differences between *V*. *sublobata* and Turkish *V*. *hederifolia* were found in six characters (Table 6). The Turkish samples differed from the European *V*. *hederifolia* samples significantly in three characters (Table 6). In all of these, these samples were more extreme than *V*. *hederifolia* in comparison with *V*. *sublobata* as seen in the biplot (Fig. 6). *Veronica sublobata* (but not European *V*. *hederifolia*) could be differentiated from Turkish *V*. *hederifolia* in the thickness of the basal element of hairs (Suppl. Figure 12) and calyx length. The three significant differences between European and Turkish *V*. *hederifolia* were also significantly different between *V*. *sublobata* and Turkish *V*. *hederifolia*: (1) the pedicel/calyx length ratio (Fig. 7), a difference caused by the length of the calyx, not the length of the pedicels, (2) larger flowers, with Turkish samples being more asymmetric, having longer but not wider flowers than European *V*. *hederifolia* (Suppl. Figures 132, 14, Table 6), and (3) the ratio of the length of the central lobe of the leaf compared to its width with the Turkish samples having the most elongated central lobe (Fig. 8; Table 6).

**Table 6:** Morphological characters from analysis of specimens including Turkish *Veronica* summarized according to taxon with standard deviation and p-value for significance (n.s. for *p* > 0.5, * for *p* < 0.01). See Suppl. Table 4 for complete measurements

Character	A) V. sublobata	B) European V. hederifolia	C) Turkish V. hederifolia	A vs. B	A vs. C	B vs. C
Leaf length [mm]	8.10 ± 1.93	9.15 ± 2.42	9.43 ± 5.02	n.s.	n.s.	n.s.
Leaf width [mm]	8.16 ± 2.74	8.74 ± 2.51	9.82 ± 6.40	n.s.	n.s.	n.s.
Central leaf lobe length [mm]	3.29 ± 0.79	4.31 ± 2.07	3.19 ± 1.20	n.s.	n.s.	n.s.
Central leaf lobe width [mm]	5.45 ± 1.68	5.02 ± 1.65	5.61 ± 2.92	n.s.	n.s.	n.s.
Central leaf lobe length/width	0.66 ± 0.21	0.83 ± 0.09	1.56 ± 0.17	*	*	*
Leaf incision depth [mm]	2.20 ± 1.07	2.27 ± 1.01	1.88 ± 0.6	n.s.	0.11	0.32
Basal hair element thickness [µm]	51.42 ± 10.16	60.05 ± 11.41	67.08 ± 13.91	0.04	*	0.24
Pedicel length [mm]	10.38 ± 3.26	12.03 ± 4.15	10.37 ± 7.27	n.s.	n.s.	n.s.
Sepal length [mm]	2.37 ± 0.66	3.32 ± 0.84	4.29 ± 1.71	*	*	0.41
Pedicel/sepal ratio	4.48 ± 1.01	3.66 ± 1.02	2.29 ± 1.03	0.03	*	*
Flower length [mm]	2.83 ± 0.7	3.49 ± 0.83	4.77 ± 1.46	0.02	*	*
Flower width [mm]	2.02 ± 0.65	2.85 ± 0.96	3.03 ± 0.83	*	*	n.s.

The results of the descriptive measurements show differences in the hairs on the calyx surface, which are lacking in most *V*. *sublobata* samples, whereas European *V*. *hederifolia* samples in almost all cases had some but very few in number, and Turkish *V*. *hederifolia* samples had almost none on the surface but were strongly ciliate (Suppl. Table 4). In addition, there is also a difference in the occurrence of hairs outside of the hairline on the pedicel, which were present often in *V*. *sublobata*, whereas in all *V*. *hederifolia* samples, this was never the case. In the number of lobes of the leaf, there was no differentiation possible between the three groups since all ranged from three to seven lobes.

## Discussion

### Species concept in *Veronica* subsect. *Cochlidiosperma*

Using a combination of DNA-based phylogenetics, flow cytometry, and morphological analyses, we sought to delimit species within *V*. subsect. *Cochlidiosperma*. Our results strongly support recognition of the five currently accepted species: *V. stewartii*, *V*. *triloba*, *V. sibthorpioides*, *V*. *sublobata*, and *V*. *hederifolia*. For Central Europe, our results confirm that the *V*. *hederifolia*-complex comprises three cytologically and taxonomically distinct entities corresponding to three ploidy levels. Diploid populations can be assigned to *V*. *triloba*, tetraploids to *V*. *sublobata*, and hexaploids to *V*. *hederifolia*, fully corroborating the conclusions of earlier studies. This clear correspondence between ploidy level and taxonomic identity supports the continued recognition of these taxa as separate species rather than as infraspecific variants. Morphological differentiation between *V*. *hederifolia* and *V*. *sublobata* remains challenging because of pronounced phenotypic plasticity, geographical variation outside Central Europe, and the limited number of non-overlapping diagnostic traits, which are not available at all developmental stages. However, our findings also suggest that taxonomic diversity within the subsection may exceed current estimates. Further research, particularly in the Eastern Mediterranean and the Near East, is needed to clarify species boundaries in these understudied regions.

At the time of their original descriptions, all species in *V*. subsect. *Cochlidiosperma* were defined on the basis of morphological characteristics, thereby adhering to a morphological species concept. In contrast to some earlier assumptions about extensive morphological overlap, all three species proved to be morphologically well distinguishable when appropriate characters are considered. The most informative trait for initial separation is the pedicel length to calyx length ratio, which reliably discriminates *V*. *triloba* by its markedly short pedicels. Subsequent differentiation between *V*. *hederifolia* and *V*. *sublobata* can be achieved using indumentum characters, particularly the arrangement of hairs on the flower pedicels (see below) and hair length on the calyx. *Veronica hederifolia* is characterized by consistently longer calyx hairs, providing a robust complementary diagnostic feature.

Among the tested multivariate approaches, LDA showed the highest discriminatory performance, with accuracy exceeding 95%, consistent with previous morphometric studies showing that LDA often matches or outperforms CART, particularly when taxa differ through combinations of continuous characters [57, 72–75]. CART was also highly discriminatory in our analyses but achieved lower accuracy. Nevertheless, it offers complementary advantages, including transparent decision rules resembling traditional taxonomic keys, fewer statistical assumptions, and suitability for exploratory analyses and practical identification [56, 57, 73]. Thus, LDA is preferable for maximizing classification accuracy, whereas CART is valuable for identifying diagnostic characters and communicating species delimitation.

To our knowledge, JRip has not previously been applied in taxonomic studies. It produced an almost ready-to-use determination key and was more interpretable than both LDA and CART. Although its accuracy was slightly above 92%, making it somewhat less discriminatory, this performance remains competitive where rule-based identification and interpretability are priorities. Future taxonomic studies should therefore consider JRip as a complementary approach alongside established methods.

An exception to the purely morphological description is *V*. *sublobata* [6] which Fischer distinguished using both morphological traits and ploidy level, reflecting an attempt to apply the biological species concept (as stated explicitly in [21]). Nevertheless, ploidy level can serve as an indirect indicator of reproductive isolation, and Fischer [6] used it as an absolute diagnostic character. The biological species concept is difficult to apply in predominantly selfing lineages such as our study species [6, 63] in which flowers are often self-pollinated before opening (but see [64]). Gadella and Kliphuis [21] observed pre-anthesis shedding of pollen in both species, whereas Benoit [64] observed it only in *V*. *sublobata*. However, Tsuruuchi [65] reported 20 different dipteran and five hymenopteran flower visitors on *V*. *hederifolia*. Ellis and Ellis-Adam [66] further included beetles as pollinators, and our own observations indicate myrmecophily as another possibility. In contrast, Müller [63] reported pseudo-cleistogamy in the species, at least in bad weather. Fischer [6] also noted stronger odor in *V*. *hederifolia* and more bee visits to *V*. *hederifolia* than to *V*. *sublobata*. Additionally, phenology may prevent crossing between the two species. We noted that *V*. *hederifolia* flowers about 2–7 days later than *V*. *sublobata*. Benoit [64] has been the only one to experimentally cross *V*. *sublobata* and *V*. *hederifolia* and did not get seed set. Our report of a triploid plant (Suppl. Tables 3, 4) is the first indication that such hybridization actually occurs, but the lack of seeds suggests that the plant was sterile. Such sterile triploids suggest the action of a triploid block, which is, however, weaker in selfers [67]. Nevertheless, there is a good chance that hybrids have been overlooked due to similarity with parents and the unclear differentiation between the parents. It is also possible that such hybrids have been overlooked even when ploidy levels were known, because hybridization can produce cytotypes corresponding to already recognized ploidy levels. For example, *V*. *triloba* and *V*. *hederifolia* could form tetraploid hybrids, triploid hybrids could form between *V*. *triloba* and *V*. *sublobata*, or autotriploid *V*. *triloba* could form hexaploids repeatedly. The occurrence of these hybrids, which may be indicated by hexaploid, triloba-like *V*. *hederifolia* in Czechia (lappago-type with glabrous calyx and smaller and more squarrose, almost triangular leaves, but hederifolia-like pedicel to calyx ratio) would need to be investigated more thoroughly.

Previous studies have also applied the ecological species criterion to differentiate the five species [4, 6, 21]. While *V. stewartii* and *V. sibthorpioides* are restricted to relictual mountain habitats, clear ecological differentiation among the remaining species is limited and it is common to find *V*. *sublobata* and *V*. *hederifolia* or *V*. *triloba* and *V*. *hederifolia* growing in one place (e.g., Suppl. Tables 3 and our samples for the growth chamber experiment). Environmental unpredictability and population sizes that often fall below habitat carrying capacity may also increase niche overlap in annuals relative to perennials [68] and lead to these common sympatric populations reported before [17, 21, 22], although others considered them to be rare [18].

In contrast, phylogenetic analyses provide valuable additional evidence for species delimitation. Molecular data not only serve as diagnostic characters but also yield insights into the parallel origins of polyploids [5, 69]. In addition, they enable rigorous testing of the diagnostic value of morphological characters within phylogenetically defined taxa. Although recent studies increasingly favor phylogenomic approaches with dense taxon sampling, we provide a preliminary framework for a more comprehensive analysis by using a single nuclear locus and a plastid locus while including all major taxa and broader sampling of the more widespread species. Together with nuclear AFLP data [5], these results are consistent with Fischer’s [6] hypothesis that *V*. *hederifolia* is an allopolyploid hybrid derived from tetraploid *V*. *sublobata* as the maternal parent and *V*. *triloba* as the paternal parent. Genome size data further support the hypothesis that *V*. *hederifolia* represents an allohexaploid derived from hybridization between *V*. *triloba* and *V*. *sublobata*. The observed genome size of *V*. *hederifolia* differs by only 1.02% from that expected for a hypothetical additive allohexaploid. Such a minor reduction is readily explained by genome downsizing, a well-documented phenomenon in newly formed allopolyploids involving the rapid loss of redundant or nonessential genomic material.

The origin of *V*. *sublobata* itself remains unresolved. Our data suggest a possible role of *V. sibthorpioides* or a close relative, as this taxon consistently appears as sister to the clade comprising *V*. *sublobata* and *V*. *hederifolia* (see Fig. 1). However, reported chromosome numbers for *V. sibthorpioides* (2n = 28 or 30; Sánchez-Agudo et al. in [70]) do not clearly correspond to any established ploidy level within the group, which otherwise has a base chromosome number of *x* = 9. This discrepancy leaves open the possibility that *V. sibthorpioides* is itself tetraploid, potentially derived from an independent origin involving at least one shared progenitor, followed by aneuploid reduction from 2n = 36 through the loss of three or four chromosomes from the haploid set.

Clarifying these relationships will require more focused studies of Mediterranean populations. Our results reveal previously unrecognized taxonomic diversity and underscore the importance of integrating phylogenetic evidence with ploidy analyses and detailed morphological analyses. In the following section, we provide a more detailed synthesis of current knowledge on morphological differentiation among the taxa of *V*. subsect. *Cochlidiosperma* before setting the scene for such more specific investigation of the Mediterranean diversity.

### Differentiation of *V*. *hederifolia* and *V*. *sublobata*

A number of different studies have previously sought to differentiate *V. hederifolia* and *V. sublobata* but in our opinion it is necessary to look at the morphology of the two species from the perspective of *V. hederifolia* being polyploid and a hybrid. We, therefore, aim to interpret the various diagnostic characters of *V*. *hederifolia* investigated by us and previous authors from these perspectives. The characters related to ploidy are pollen size and stomata size [6, 17, 64] (Suppl. Table 1). The idea to consider hair size came in recent years, when Michael Wilcox (pers. comm.) highlighted the length of the first segment of the hairs on the side of the calyx to be much greater in *V. hederifolia* (250–350 microns) than in *V*. *sublobata* (150–200 microns). In parallel, Bomble [22] also noted that the hairs on the calyx of *V*. *hederifolia* were thicker than those of *V*. *sublobata*. The size of the basal element of the hair, indeed, seems to be different between the two species (Table 6) and may be influenced by ploidy variation [71]. Probably, it is not just the size of the basal element that is related to ploidy but also the complete trichome size on leaf and calyx, which was already suggested by Bomble [22] to be a good character to distinguish the species, as confirmed here (Suppl. Figure 3).

In addition, several other characters may exhibit a ploidy-related gigas effect, including flower size [6], although this trait may also be influenced by environmental conditions. Style and anther length, which Gadella and Kliphuis [21] regarded as the most reliable characters for species differentiation, may likewise reflect this effect. Bomble [22] further identified seed size as a useful character for distinguishing the two species. Across all of these traits, *V*. *hederifolia* exhibits larger dimensions than any of its close relatives.

According to Rieseberg and Ellstrand [72], more than a third of the characters of a hybrid species are intermediate between its parents, and several such intermediate characters can be found in *V*. *hederifolia*. It was already emphasized by Fischer [4] that the species can vary considerably in morphology depending on the environment, but *V*. *hederifolia* always stays intermediate between its parents in a particular habitat. However, also on a larger scale, *V*. *hederifolia* is intermediate in many morphological characters because of intermediate habitat requirements with respect to light and water.

One such character, commonly used in floras [29, 73], is the pedicel/calyx length ratio. As shown by our results, the difference in the ratio between the species is caused by calyx length, not pedicel length, which is modifiable by environment [4, 17] and also makes the ratio plastic [22]. We found a large overlap between the two species in the range of 2.5 to 5.0 and, therefore, one should not rely on this character alone except in extreme cases.

Also, the pedicel indumentum is more or less intermediate. All three Central European species have a prominent row of short hairs adaxially on the pedicel. Only *V*. *sublobata* may exhibit hairs arranged around the entire pedicel circumference, although this character is unilateral because the species may also show a single-row arrangement. This is likely an intermediate character for *V*. *sublobata* combining the clear line of hairs from *V*. *triloba* (assuming it or a similar ancestor to be one ancestor of *V. sublobata*) with the hairs all around the pedicel from *V. sibthorpioides* or its ancestor. However, the number of hairs is to some degree variable between populations of both *V*. *sublobata* and *V*. *hederifolia* [6, 17].

Another intermediate character is flower color. *Veronica triloba* is typically characterized by dark blue flowers, whereas *V*. *sublobata* most often exhibits pinkish-white flowers. Flower color in *V*. *hederifolia* is intermediate, with various shades of blue prevailing, which is consistent with its inferred hybrid origin. However, flower color can be misleading, particularly when assessed from field photographs under variable lighting conditions, and should not be used as a sole diagnostic character. Generally, flower color has been considered a highly reliable character before [4] with only rarely white flowers encountered in *V*. *hederifolia* (Schütz, pers. obs.). Nevertheless [17] noticed genetic variation within species and [22] reported that flowers of *V*. *hederifolia* get darker in wet-cold weather. Again, in such cases both species may vary in the same direction, so that the character is useful in sympatric populations but not always in single-species populations.

The same applies to the calyx indumentum. *Veronica triloba* is characterized by pubescent calyces, whereas *V*. *sublobata* has glabrous calyces, at least in most of the cases, and *V*. *hederifolia* has scattered hairs, especially in the lower part [6]. Nordenstam & Nilsson [17] reported genetic variation in this character in Scandinavia. We did not investigate this character systematically but largely confirmed the pattern observed by Fischer [6]. Similar intermediacy in *V*. *sublobata* is found in seed shape, which is oblong and distinctly ribbed in *V*. *triloba* and suborbicular and shallowly ribbed in *V*. *sublobata* [4].

Gigas effect and hybrid intermediacy play a lesser role in the leaves. The whole subgenus is characterized by the prostrate habit and reduction of the vegetative part compared to the reproductive part. The lack of photosynthetic tissue in the vegetative part is compensated by an increase in leaf area, which consequently resembles leaves. A number of characters in leaves have been used to differentiate the two taxa (Table 1, Suppl. Table 1). Elliptic Fourier analyses revealed extensive overlap in leaf outlines among all three species (Suppl. Figure 7, 12), rendering leaf blade shape alone unsuitable for reliable identification. This finding highlights the importance of quantitative evaluation of traditionally used characters and underscores that not all visually prominent traits contribute meaningfully to taxonomic resolution within the *V*. *hederifolia* complex. Our experiments further demonstrate the reason why this has not been possible. Fischer [6] reported that the variation within populations is low but between populations is high. Our results suggest that it is also the intraindividual variation that causes problems. For example, the central lobe was stated by Fischer [6] to be longer than wide in *V*. *sublobata* but the opposite in *V*. *hederifolia* as confirmed by [17] but disputed by [21]. We, however, noted a considerable positional effect with upper leaves having an elongated central lobe. A positional effect was already noted by Fischer [6] but not described. While this positional effect is likely to be caused by light availability, we did not see such an effect in our light treatments. A similar effect is seen in the leaf incision, which, as the name suggests, was reported to be lower in *V*. *sublobata* [6]. In our analysis the lower leaves are less notched than the upper ones. Leaf incision reduces leaf area and is, therefore, likely of lesser advantage in the shadier habitat of *V*. *sublobata*, although we did not see, again, an effect in our treatments (results not shown). Large variation, with a major influence of position blurring differences between the species, is also seen in the number of lobes (Suppl. Figure 5). However, in general there is again intermediacy, with *V*. *hederifolia* having more lobes than *V*. *triloba* and less than *V*. *sublobata*. Intermediacy was also noted by Fischer [6] in petiole length, although [17] called it a very plastic character. Indeed, Dale [74] demonstrated increased leaf length in shaded *V. montana*. Our results show the same trend (Suppl. Figure 8) with short petioles associated with positive PC1 and PC2 found in the light treatment and upper leaves. A similar trend is found in the trichome density on the leaves, which is higher in the dark and the shade-adapted *V*. *sublobata* than in the light and *V*. *hederifolia*, again without the possibility of clearly differentiating the two species using this character (Fig. 3b, c). Therefore, the intermediacy of these characters noted before seems only to follow the intermediacy in ecological preferences of the species.

Finally, a gigas effect and an ecological effect may be responsible for the difference in color and thickness of the leaves. Leaves in shadier habitats are known to have higher SLA [75], which is fully supported by our results (Fig. 3). Additionally, the difference between *V*. *hederifolia* and *V*. *sublobata* also suggests that *V*. *sublobata* is more adapted to shadier habitats and confirms previous observations that leaves of *V*. *hederifolia* are fleshier and more succulent [6, 21, 64]. However, the overlap due to treatment is larger than the interspecies differences precluding the use of this character to differentiate species other than in mixed populations. It is less clear how leaf color is influenced. Values in red and green light are higher for *V*. *sublobata* than for *V*. *hederifolia* (Suppl. Figure 6) supporting the observations of others [6, 21, 64] that leaves of *V*. *hederifolia* are darker than those of *V*. *sublobata*. A likely explanation is chlorophyll content, which increases with ploidy [76, 77]. Buono and Albach [77] found more chlorophyll in *V*. *longifolia* and *V*. *spicata* with higher ploidy. Darker leaves in light, as seen in our treatments (Suppl. Figure 6), are, however, in contrast to other studies showing increases in chlorophyll in the shade [78]. So, it is likely that other compounds also influence the color of leaves, but darker leaves in *V. hederifolia* are a good way to differentiate the two species in sympatric populations, as suggested by previous authors (Suppl. Table 1).

### Mediterranean lineages

With the species delimitation in Central Europe mostly clarified, we can focus on southeastern Europe, Anatolia and the Near East where species delimitation is less clear as indicated by our samples from southern Türkiye. Fischer [79] already mentioned that the variability in *V*. *hederifolia* increases from northwest to southeast and that the corolla size and style length decrease towards the southeast. We have included three samples, of which the two samples from southern Türkiye were collected within 200 m of each other. Unfortunately, no ploidy is known for these three samples. The sample from Cyprus shows a pattern indicative of hybridization between *V*. *triloba* and *V*. *sublobata* or *V*. *hederifolia* based on its incongruent placement between nuclear ITS and plastid DNA analyses (Fig. 1). We cannot further discuss the sample because the voucher is rather small and appears to be typical *V*. *hederifolia* and characters such as flowers are not visible.

The two samples from Türkiye show a completely different pattern being related to the Western Mediterranean *V. sibthorpioides* in the plastid DNA analysis but more closely related to *V*. *triloba* in nuclear ribosomal DNA. The relation to *V*. *triloba* and *V. sibthorpioides* becomes morphologically apparent in the lower pedicel/calyx length ratio. Intermediates between *V*. *hederifolia* and *V*. *triloba* have been reported from the Southern Balkans before [6]. However, the specimens from southern Türkiye differ from *V*. *triloba* and *V. sibthorpioides* in having larger corollas, which are more asymmetric (longer than wide), longer calyces, which are strongly ciliate and have no hairs on the surface (differentiating them from *V*. *triloba* but not *V. sibthorpioides*). Conspicuous is the elongated central lobe of the leaf (Table 6), which is typical for plants and leaves that get more light based on our growth chamber experiment (Suppl. Figure 4). These plants also differ clearly from *V*. *hederifolia* f. *nana* Halacsy, small and little-branched plants, which are densely hairy and have pedicels with hairs all around the pedicel. These have been reported from Greece and Anatolia [6] but only testify to the large morphological variation in the eastern Mediterranean region that is taxonomically insufficiently accounted for so far.

Morphological variation is also visible in specimens of *V*. *hederifolia* from Crimea and southern Russia as well as in Iran. However, the latter are clearly associated with *V*. *hederifolia* (Fig. 1). Also here, inference of ploidy along with DNA sequence analysis and morphological investigation is warranted given reports of typical but tetraploid *V*. *hederifolia* from Iran [80].

### Concluding remarks

Taken together, our analyses provide strong evidence for the recognition of three species in Central Europe and underscore the need for further investigation of populations from the Mediterranean and Asia, where diversity is likely underappreciated. These investigations necessarily require parallel analyses of DNA sequence data, ploidy levels, and morphological characters. However, care needs to be taken to analyze stable characters that are not influenced by the environment. As our growth chamber experiments revealed, light is the more important factor influencing the phenotype of these plants than water.

## Supplementary information

Below is the link to the electronic supplementary material.

Supplementary Material 1

Supplementary Material 2

Supplementary Material 3

Supplementary Material 4

Supplementary Material 5

Supplementary Material 6

Supplementary Material 7

Supplementary Material 8

Supplementary Material 9

Supplementary Material 10

Supplementary Material 11

Supplementary Material 12

Supplementary Material 13

Supplementary Material 14

Supplementary Material 15

Supplementary Material 16

Supplementary Material 17

Supplementary Material 18

Supplementary Material 19

Supplementary Material 20

Supplementary Material 21

Supplementary Material 22

## Data Availability

The DNA sequence data supporting the conclusions of this article is available under the GenBank accession numbers given in Table 2. All other data supporting the conclusions of this article is included within the article and its additional files.
