# Supplementary material for: Species limits in Veronica hederifolia and relatives: experimental, morphometric, and phylogenetic approaches in a weedy polyploid complex
Source: BMC Plant Biol. 2026 Oct 22;26:1819. doi: 10.1186/s12870-026-10212-6 (PMC13692848; doi:10.1186/s12870-026-10212-6)
Supplement: Supplementary file 1 [file 12870_2026_10212_MOESM16_ESM.docx]

Supplementary Tables

Suppl. Table 1: Characters used to distinguish *V. hederifolia*/*V. sublobata* based on morphology in previous publications and in the current study.

| Character | Fischer (1967) | Nordenstam & Nilsson (1969) | Benoit (1971) | Gadella & Kliphuis (1976) | Bomble (2015)  Literature summary | This study |
| --- | --- | --- | --- | --- | --- | --- |
| Leaf fleshiness | weakly succulent/ not succulent |  | more robust and fleshy/ Less robust | mostly subcarnose/ mostly thin, herbaceous | thick, stiff /thin, soft | (31-)51-126 mm2/ mg/ 37-80 mm2/ mg |
| Leaf color | medium green/ Light green |  | Greyish green/ bright green | dark green/ Light to yellowish green | dark green/ Light green (to yellowish green | Difference measurable |
| Leaf lobes | 3-5 /5(-7) | 3(-5)/ 5 |  | 3-5/3-5(-8) | 3-5 /(3-)5(-7)/ | 2-7/3-8 |
| Leaf incision | deeply lobed/ Shallowly lobed | Deep/shallow/ | Deeper/ shallower |  |  | 0.51-0.83 mm/ 0.55-1.34 mm |
| Central lobe [length/ width] | A bit longer than wide/ A bit wider than long | Wider than long/ a bit wider than long |  | In both wider than long | 0.9-1.1/ 0.7-0.9 | (0.27-)0.59-0.83(-0.89)/ (0.29-)0.55-1.34 |
| Lobe apex | Obtuse to rounded/ obtuse with acute tip | Obtuse to rounded/ obtuse to subacute | Obtuse/ more acute | Sometimes acute/ mostly acute |  |  |
| Leaf incision | Obtuse/ acute |  |  | Obtuse and acute in both |  | Length lamina/ length incision: (4.2-)6.1-17.6/ (2.1-)4.2-43.8 |
| Trichome density adaxial [/ mm^2^] |  |  |  |  |  | 0.44-5/ (0.0-)0.5-2.9 |
| Trichome length adaxial [µm] |  |  |  |  |  | 0.15-0.39(-0.5)/ 0.29-0.56 |
| Stomata size [µm] | 33-36/ 38-43 |  | 26-35/35-48 |  |  |  |
| Pedicel/ calyx ratio | 3.5-7/ 2-4 | 3.5-4.5/ 1.5-2 | 4-5/ 2.5-3.5 | 2-6/ 2-3(-6) | 3-7/ 2-4 | (1.1-)2.8-6.8(-8.7)/  (1.1-)1.9-5.4 |
| Pedicel indumentum apart from central row | more or less scattered, spreading hairs/ very few | None to densely scattered hairs/ none to few scattered hairs |  | 54%/ 5% with scattered hairs | Often present/ almost always lacking | Several to many/ few to several |
| Calyx surface | Scattered hairs to glabrous/ Glabrous | Scattered hairs to glabrous/ few hairs to glabrous |  | 95%/ 100% glabrous | Mostly lacking/ mostly few |  |
| Calyx cilia [mm] | 0.5-0.9/ 0.9-1.2 | Shorter/ longer |  | 91%/ 7% with hairs 0.5-0.9 | 0.5-0.9/ 0.7-1.2 |  |
| Calyx cilia #, thickness |  |  |  |  | 25-35, thin/ 30-40, thick | 36-71µm/ 44-83µm |
| Corolla diameter [mm] | 4-6/ 6-9 | Petal length: 1.8-3.0/ 2.4-2.8 | Corolla length: 2/ 2.5-3 | Highly reliable | 4-6/ 5-7 | Corolla length 1.6-3.4(-4.7)/ 2.2-5.9 |
| Corolla color | Pale lilac to white/ bright blue with clear white center | Pale lilac to white/ Light blue to white | Pale lilac to whitish/ blue | Highly reliable | Whitish to pale lilac/ white to light blue or lilac with clear white center | White to light blue/ violet with white center, rarely white |
| Corolla nerves | 16-19/ 20-22 |  |  | Highly reliable | 14-19/ 20-22 |  |
| Anther length [mm] | 0.4-0.8/ 0.7-1.2 |  | 0.6/ 1 | 94%/ 92% matching Fischer (1967) | 0.4-0.8/ 0.7-1.2 |  |
| Anther color | Light blue to pale/ azure blue |  | Whitish/ bright blue | See above | White to light blue/ azure blue |  |
| Style length [mm] | 0.3-0.5/ 0.7-1.1 | 0.3-0.8/ 0.6-1.2 |  | See above | (0.2-)0.3-0.6(0.7)/ (0.6-) 0.7-0.9(-1.1) |  |
| Seed size length x width [mm] | 2.5 x 2.1/ 2.8 x 2.3 |  |  | 33% 2.5-3 long/ 35% 2-2.5 long | 2.2-2.7 x 2.0-2.4/ 2.6-3.1 x 2.2-2.7 |  |
| Seed cavity mouth | Narrow with clear ring/ intermediate without ring |  |  | As in Fischer (1967) | Whitish ring/ as rest of seed |  |
| Seed color | Rust-brown/ yellowish brown |  |  | 81%:19% brown:yellow/ 40%:60% | Reddish brown/ light yellow |  |
| Pollen size [µm] | 33-37/ 36-43 | 31-38/ 36-40 | 31-35/ 38-42 |  |  |  |

Suppl. Table 2: Genome size measurements known for *V*. sect. *Cochlidiosperma* (other than Table 4 from Czech Rep.). 1C – holoploid genome size (genome size per nucleus in pg), 1Cx – monoploid genome size (genome size per chromosome complement in pg), CV – coefficient of variation, superscript indicating standard used, ^1^ – *Solanum pseudocapsicum*, ^2^ – *Pisum sativum*, 3 – *Hedychium gardnerianum*.

| Species | Ploidy | 1C-value | 1Cx-value | number of individuals | CV | Country, Locality | Voucher | Source |
| --- | --- | --- | --- | --- | --- | --- | --- | --- |
| *V. hederifolia* | 6 | 1.88 | 0.315 |  | 1,46 | Czech Republic, Moravia, Drásov (distr. Brno-venkov) |  | Šmarda et al. 2019 |
| *V. hederifolia* | 6 | 2.08 | 0.347 | 1 |  |  |  | Castro et al. 2012 |
| *V. hederifolia* | 6 | 2.013 | 0.336 | 1 | 4.2^1^ | Germany, Hessia, Taunus TK6013211 | Wittig, OLD |  |
| *V. hederifolia* | 6 | 2.025 | 0.338 | 1 | 2.7^1^ | France, Lautaret, 2100 m | Albach S1018, OLD | initially determined as *V. triloba* |
| *V. hederifolia* | 6 | 2.059 | 0.343 | 1 | 1.9^1^ | Germany, Hessia, Schlangenbad- | Rüdiger Wittig 7, OLD05799 |  |
| *V. hederifolia* | 6 | 2.06 |  |  |  |  |  | Zonneveld 2019 under *V. sublobata* |
| *V. hederifolia* | 6 | 2.063 | 0.344 | 1 | 4.2^1^ | Germany, Hessia, Siedlung westl. Nonnenmühle | Rüdiger Wittig 3, OLD05798 |  |
| *V. hederifolia* | 6 | 2.068 | 0.345 | 1 | 1.8^1^ |  | Albach 2073, OLD |  |
| *V. hederifolia* | 6 | 2.072 | 0.345 | 1 | 4.6^1^ | Germany, Hessia, Taunusstein-Hahn | Rüdiger Wittig 2 |  |
| *V. hederifolia* | 6 | 2.077 | 0.346 | 1 | 2.2^1^ | Germany, Hessia, Schlangenbad-Bärstadt | Rüdiger Wittig 1 |  |
| *V. hederifolia* | 6 | 2.113 | 0.352 | 1 | 4.8^2^ | Germany, Rhineland-Palatia, Trifels | Albach 1504, OLD05781 |  |
| *V. hederifolia* | 6 | 2.134 | 0.356 | 3 | 2.40-3.41^1^ | Germany, Northrhine-Westphalia, Münster | Albach S842, OLD |  |
| *V. hederifolia* | 6 | 2.199 | 0.367 | 1 | 4.8^1^ | Germany, Lower Saxony, Conneforde | Albach 1507, OLD05763 |  |
| *V. hederifolia* s.l. | 4 | 1.3-1.4 |  | 1 | >5^1^ | Turkey, Antalya: 5-10 km west of Ibradi, towards Gembos Plateau | Albach 1080, OLD00548 |  |
| *V. sublobata* | 4 | 1.295 | 0.325 |  | 0.81 | Czech Republic, Moravia, Dolní Dunajovice (distr. Břeclav): Dunajovické kopce Nature Reserve |  | Šmarda et al. 2019 |
| *V. sublobata* | 4 | 1.407 | 0.352 | 3 | 1.7 | Germany, Northrhine-Westphalia, Bonn-Bad Godesberg, Waldkrankenhaus | Albach 1498, OLD06046 |  |
| *V. sublobata* | 4 | 1.410 | 0.353 | 5 | 2.6 | Austria, Vienna, weedy in Botanical Garden | Albach 589, WU | Albach & Greilhuber 2004 |
| *V. sublobata* | 4 | 1.427 | 0.357 | 3 | 1.8-3.8 | Germany, Northrhine-Westphalia, Cologne | Albach 1499, OLD |  |
| *V. sublobata* | 4 | 1.435 | 0.359 | 3 | 2.8-3.4 | Germany, Northrhine-Westphalia, Münster, Gelmer Kanalufer | Albach S843, OLD |  |
| *V. sublobata* | 4 | 1.441 | 0.360 | 1 | 2.1 | Germany | Rüdiger Wittig, Palette 24, OLD05766 |  |
| *V. sublobata* | 4 | 1.445 | 0.361 | 1 | 2.7 | Germany, Hessia, Siedlung westl. Nonnenmühle | Rüdiger Wittig 9 |  |
| *V. sublobata* | 4 | 1.453 | 0.363 | 1 | 2.5 | Germany, Hessia, Taunusstein-Bleidenstadt | Rüdiger Wittig 8 |  |
| *V. sublobata* | 4 | 1.461 | 0.365 | 3 | 2.3-3.8 | Germany, Lower Saxony, Oldenburg, Alexandersfeld | Albach 1497, OLD06047 |  |
| *V. sublobata* | 4 | 1.461 | 0.365 | 1 | 1.9 | Germany, Hessia: Oberursel, Maasgrundweiher 5717/344 | Rüdiger Wittig Palette 10, OLD05765 |  |
| *V. sublobata* | 4 | 1.462 | 0.366 | 3 | 2.7-3.2 | Austria, Lower Austria, Waldviertel, Persenbeug, Hofamt Priel, Fürholzstrasse | Albach S844, OLD |  |
| *V. sublobata* | 4 | 1.472 | 0.368 | 1 | 1.9 | Germany, Hessia: Schneidhain, Sportgelände, 5816/232 | Rüdiger Wittig Palette 5 |  |
| *V. sublobata* | 4 | 1.476 | 0.369 | 1 | 4.2 | Germany, Northrhine-Westphalia, Olsberg | Albach 1500, OLD05782 |  |
| *V. sublobata* | 4 | 1.478 | 0.370 | 1 | 4.1^3^ | Switzerland, Bern: Blumenbergstr. 51 | Muriel Bendel no. 2/2020, OLD08473 |  |
| *V. sublobata* | 4 | 1.481 | 0.370 | 1 | 4.0 | Germany, Rhineland-Palatia, Mainz – University Campus | Albach 1508, OLD05789 |  |
| *V. sublobata* | 4 | 1.482 | 0.371 | 3 | 0 | Germany, Lower Saxony, Oldenburg, weedy in Botanical Garden | Bauer 1.5.2011, OLD00358 |  |
| *V. sublobata* | 4 | 1.489 | 0.372 | 3 | 3.2-4.8 | Germany, Northrhine-Westphalia, Sendenhorst Albersloh | Albach S841, OLD |  |
| *V. sublobata* | 4 | 1.490 | 0.373 | 3 | 3.2-4.3 | Austria, Salzburg, Tennengau Hallein, Kuchl | Albach S857, OLD05846 |  |
| *V. sublobata* | 4 | 1.493 | 0.373 | 1 | 4.6 | Germany, Hessia,  Schlangenbad | Rüdiger Wittig 5 |  |
| *V. sublobata* | 4 | 1.502 | 0.376 | 1 | 1.9 | Germany, Oldenburg, Nedderend | Albach 2061, OLD15615 |  |
| *V. sublobata* | 4 | 1.506 | 0.377 | 1 |  | Germany, Taunus TK6013211 | Wittig OLD |  |
| *V. sublobata* | 4 | 1.514 | 0.379 | 1 | 4.8 | Germany, Rhineland-Palatia, Trier, Speestr. | Albach 1502, OLD05785 |  |
| *V. sublobata* | 4 | 1.520 | 0.380 | 3 | 4.7-5.4 | Germany, Saxony, Herrenhut | Albach S840, OLD |  |
| *V. sublobata* | 4 | 1.529 | 0.382 | 1 | 4.1^1^ | Switzerland, Bern: Papiermühlenstraße | Muriel Bendel no. 1/2020, OLD08472 |  |
| *V. sublobata* | 4 | 1.549 | 0.387 | 1 | 2.2 | Germany, Rhineland-Palatia, Idar-Oberstein | Albach 1503, OLD05784 |  |
| *V. sublobata* | 4 | 1.549 | 0.387 | 1 | 6.6 | Germany, Hessia, Königstein, Opel-Zoo | Albach 1505, OLD05764 |  |
| *V. sublobata* | 4 | 1.55 |  |  |  |  |  | Zonneveld 2019 under *V. hederifolia* |
| *V. sublobata* | 4 | 1.609 | 0.402 | 1 | 10.8^1^ | Germany, Saxony, Dresden, Reichenbachstr./Teplitzer Str. | Albach 1326, OLD00399 |  |
| *V. triloba* | 2 | 0.612 | 0.306 | 4 | 3.1 | Turkey, Troy | Albach 232, WU | Albach & Greilhuber 2004 |

Suppl. Table 3: Information on samples analyzed in the detailed Czech study of ploidy distribution. Individual based information on genome size measurements are found in Suppl. Table 3. Voucher specimens were deposited at the Department of Biology, Faculty of Science, Jan Evangelista Purkyně University in Ústí nad Labem, Czech Republic. Populations marked with an asterisk were used in the morphometric analysis.

| Population | 2x | 3x | 4x | 6x | Latitude | Longitude | Date | Locality |
| --- | --- | --- | --- | --- | --- | --- | --- | --- |
| 001/16 |  |  | 1 |  | 49.42884 | 14.83174 | 22.04.2016 | Czechia, distr. Tábor, Dolní Hořice: edge of a crop field at the S foot of the Pacova hora Hill |
| 003/16 |  |  |  | 1 | 49.57340 | 14.33126 | 22.04.2016 | Czechia, distr. Příbram, Petrovice: edge of a crop field near the Divišova jeskyně Cave |
| 010/16 |  |  | 1 | 1 | 48.82181 | 14.28091 | 23.04.2016 | Czechia, distr. Český Krumlov, Staré Dobrkovice: shrubs NW of the village |
| 011/16 | 1 |  | 1 |  | 49.27793 | 13.94043 | 24.04.2016 | Czechia, distr. Strakonice, Řepice: edge of a crop field at the E edge of the village |
| 014/16 |  |  | 1 |  | 49.30779 | 13.96355 | 24.04.2016 | Czechia, distr. Strakonice, Osek: edge of a crop field S of the village |
| 017/16 |  |  | 1 |  | 49.25892 | 13.87251 | 25.04.2016 | Czechia, distr. Strakonice, Strakonice: deciduous forest in the pheasantry between Strakonice and Pracejovice |
| 018/16 |  |  | 1 |  | 49.26610 | 13.83792 | 25.04.2016 | Czechia, distr. Strakonice, Katovice: alluvial forest by the Otava River |
| 021/16 |  |  | 1 |  | 49.27309 | 13.61822 | 25.04.2016 | Czechia, distr. Klatovy, Rabí: alluvial forest by the Otava River, S of the town |
| 022/16 |  |  | 1 |  | 49.27280 | 13.61885 | 25.04.2016 | Czechia, distr. Klatovy, Rabí: alluvial forest by the Otava River, S of the town |
| 023/16 |  |  | 1 | 1 | 48.87924 | 16.66488 | 30.04.2016 | Czechia, distr. Břeclav, Pavlov: edge of a crop field NW of the village |
| 024/16 | 1 |  | 1 | 1 | 48.82603 | 16.64180 | 30.04.2016 | Czechia, distr. Břeclav, Klentnice: steppe vegetation in the Kočičí skála nature reserve |
| 025/16 |  |  | 1 |  | 48.82212 | 16.64066 | 30.04.2016 | Czechia, distr. Břeclav, Klentnice: steppe vegetation in the N part of the Turold nature reserve |
| 026/16 |  |  | 1 |  | 48.78203 | 16.71707 | 01.05.2016 | Czechia, distr. Břeclav, Sedlec: edge of a crop field E of the village |
| 034/16 |  |  | 1 | 1 | 49.58331 | 14.33861 | 22.04.2016 | Czechia, distr. Příbram, Týnčany: edge of a crop field 430 m E (azimuth 87°) of the chapel in the village center |
| 035/16 |  |  | 1 | 1 | 49.50883 | 14.07186 | 22.04.2016 | Czechia, distr. Písek, Horosedly: crop field 1.55 km ESE (azimuth 113°) of the chapel in the village |
| 036/16 |  |  | 1 | 1 | 48.91472 | 14.48862 | 23.04.2016 | Czechia, distr. České Budějovice, Plav: field margin 1.49 km N (azimuth 359°) of the chapel in the village center |
| 037/16 | 1 |  | 1 | 1 | 48.82730 | 14.33045 | 23.04.2016 | Czechia, distr. Český Krumlov, Domoradice: disturbed place next to railway track 310 m WbS (azimuth 257°) of the Domoradice railway station |
| 038/16 | 1 |  | 1 |  | 48.85057 | 14.22203 | 23.04.2016 | Czechia, distr. Český Krumlov, Chvalšiny: field margin at the SW slope of the spot height of 630 m a.s.l., 880 m ESE (azimuth 116°) of the church in the village center |
| 039/16 |  |  | 1 | 1 | 48.85221 | 14.22239 | 23.04.2016 | Czechia, distr. Český Krumlov, Chvalšiny: boundary between forest and crop field at the W slope of the spot height of 630 m a.s.l., 840 m EbS (azimuth 104°) of the church in the village center |
| 040/16 |  |  | 1 |  | 48.82181 | 14.28091 | 23.04.2016 | Czechia, distr. Český Krumlov, Staré Dobrkovice: shrubs at the S slope of the Nad Mlýnem Hill, 1.64 km WNW (azimuth 291°) of the petrol station in the village |
| 041/16 |  |  | 1 | 1 | 49.27793 | 13.94043 | 24.04.2016 | Czechia, distr. Strakonice, Řepice: field thicket 490 m SEbE (azimuth 125°) of the church in the village center |
| 042/16 | 1 |  | 1 | 1 | 49.29217 | 13.94379 | 04.06.2016 | Czechia, distr. Strakonice, Rovná: field at the W slope of the Zbuš Hill 970 m NWbW of the chapel in the village center |
| 043/16 |  |  |  | 1 | 49.30783 | 13.96353 | 24.04.2016 | Czechia, distr. Strakonice, Rohozná: edge of a crop field 690 m S (azimuth 184°) of the chapel (next to the chateau) in the village center |
| 044/16 | 1 |  |  | 1 | 49.30779 | 13.96355 | 24.04.2016 | Czechia, distr. Strakonice, Rohozná: edge of a crop field 690 m S (azimuth 184°) of the chapel (next to the chateau) in the village center |
| 045/16 | 1 |  | 1 | 1 | 49.29841 | 13.93031 | 24.04.2016 | Czechia, distr. Strakonice, Domanice: edge of a crop field 660 m EbS (azimuth 99°) of the chapel in the village center |
| 046/16 | 1 |  | 1 | 1 | 49.29018 | 13.85878 | 24.04.2016 | Czechia, distr. Strakonice, Krty-Hradec: verge of an agricultural road 730 m ESE (azimuth 109°) of the chapel in the village center |
| 049/16 | 1 | 1 | 1 | 1 | 49.31232 | 13.73466 | 25.04.2016 | Czechia, distr. Klatovy, Horažďovice: field at the S slope of the Na Hrádku Hill 2.67 km ESE (azimuth 117°) of the church in the town center, OLD09942 |
| 050/16 | 1 |  | 1 | 1 | 49.30389 | 13.70029 | 25.04.2016 | Czechia, distr. Klatovy, Boubín: edge of a crop field 460 m NE (azimuth 42°) of the chapel in the village center |
| 051/16 |  |  | 1 |  | 49.29861 | 13.66977 | 25.04.2016 | Czechia, distr. Klatovy, Velké Hydčice: edge of a crop field 140 m SEbE (azimuth 126°) of the chapel in the village center |
| 053/16 |  |  | 1 | 1 | 48.87924 | 16.66488 | 30.04.2016 | Czechia, distr. Břeclav, Pavlov: edge of a vineyard 700 m NWbN (azimuth 323°) of the church in the village center |
| 056/16 |  |  | 1 |  | 48.78203 | 16.71707 | 01.05.2016 | Czechia, distr. Břeclav, Sedlec: edge of a crop field 1.44 km WbS (azimuth 78°) of the church in the village center |
| 057/16 |  |  | 1 |  | 48.78556 | 16.71584 | 24.07.2016 | Czechia, distr. Břeclav, Sedlec: field 1.46 km NEbE (azimuth 62°) of the church in the village center |
| 059/16 |  |  | 1 |  | 48.89038 | 16.78399 | 01.05.2016 | Czechia, distr. Břeclav, Zaječí: crop field 190 m NEbE (azimuth 56°) of the Zaječí railway station |
| 063/16 |  |  | 1 | 1 | 48.81868 | 16.77889 | 01.05.2016 | Czechia, distr. Břeclav, Nejdek: disturbed place in a meadow 780 m NNE (azimuth 21°) of the chapel in the village center |
| 068/16 |  |  | 1 | 1 | 48.89523 | 15.91773 | 02.05.2016 | Czechia, distr. Znojmo, Milíčovice: sparse tree plantation 1.25 km WbN (azimuth 287°) of the church in the village center |
| 005/17 | 1 |  | 1 |  | 48.90700 | 16.05048 | 10.04.2017 | Czechia, distr. Znojmo, Únanov: edge of a crop field 1.24 km NWbN (azimuth 303°) of the church in the village center |
| 007/17 |  |  | 1 |  | 48.87233 | 16.08784 | 10.04.2017 | Czechia, distr. Znojmo, Kuchařovice: field margin 970 m ESE of the chapel in the village center |
| 008/17 | 1 |  | 1 |  | 48.78629 | 16.01523 | 10.04.2017 | Czechia, distr. Znojmo, Šatov: edge of a crop field 1.01 km SEbS (azimuth 149°) of the church in the village center |
| 009/17 |  |  | 1 | 1 | 48.82249 | 16.12794 | 10.04.2017 | Czechia, distr. Znojmo, Načeratice: edge of a crop field 1.19 km ENE (azimuth 69°) of the church in the village center |
| 013/17* | 1 |  |  |  | 48.87509 | 16.66110 | 11.04.2017 | Czechia, distr. Břeclav, Pavlov: dry grassland on the S slope of the Dívčí hrad Hill |
| 022/17* |  |  | 1 | 1 | 48.86813 | 16.66180 | 11.04.2017 | Czechia, distr. Břeclav, Pavlov: ditch at the edge of a crop field 930 m SW (azimuth 224°) of the church in the village center |
| 025/17* |  |  | 1 | 1 | 48.76524 | 16.78653 | 12.04.2017 | Czechia, distr. Břeclav, Hlohovec: edge of a crop field 2.10 km SE (azimuth 130°) of the church in the village center |
| 027/17* | 1 |  | 1 |  | 48.76666 | 16.78129 | 12.04.2017 | Czechia, distr. Břeclav, Hlohovec: edge of a crop field 1.71 km SE (azimuth 134°) of the church in the village center |
| 028/17 | 1 |  | 1 |  | 48.83910 | 16.73781 | 12.04.2017 | Czechia, distr. Břeclav, Bulhary: edge of a crop field 1.14 km NWbN (azimuth 330°) of the church in the village center |
| 040/17* | 1 |  | 1 |  | 50.54612 | 14.07013 | 24.04.2017 | Czechia, distr. Litoměřice, Malíč: edge of a crop field 890 m WNW (azimuth 291°) of the bus station in the village center |
| 042/17* | 1 |  | 1 |  | 50.45622 | 14.17783 | 24.04.2017 | Czechia, distr. Litoměřice, Doksany: crop field 1.36 km EbN (azimuth 84°) of the chateau in the town |
| 046/17 |  |  | 1 |  | 50.44775 | 14.29295 | 24.04.2017 | Czechia, distr. Litoměřice, Kyškovice: alluvial forest 540 m EbS (azimuth 103°) of the bus station in the village |
| 047/17* |  |  | 1 | 1 | 50.13162 | 16.14528 | 25.04.2017 | Czechia, distr. Rychnov nad Kněžnou, Čestice: edge of a crop field 630 m NbE (azimuth 11°) of the chapel in the village center |
| 048/17 |  |  | 1 | 1 | 50.14293 | 16.12595 | 25.04.2017 | Czechia, distr. Rychnov nad Kněžnou, Rašovice: edge of a crop field 580 m SWbS (azimuth 215°) of the chapel in the village center |
| 050/17 | 1 |  | 1 | 1 | 50.19615 | 16.09594 | 25.04.2017 | Czechia, distr. Rychnov nad Kněžnou, Bolehošť: edge of a crop field 1.44 km SSE (azimuth 154°) of the chateau in the village |
| 051/17* | 1 |  | 1 | 1 | 50.23522 | 16.07543 | 25.04.2017 | Czechia, distr. Rychnov nad Kněžnou, Očelice: edge of a crop field 760 m SE (azimuth 130°) of the chapel in the village center |
| 052/17* |  |  | 1 |  | 50.27561 | 14.36395 | 03.05.2017 | Czechia, distr. Mělník, Zlosyň: edge of a crop field 370 m SE (azimuth 231°) of the chapel in the village center |
| 054/17* | 1 |  | 1 | 1 | 49.30618 | 13.93885 | 08.05.2017 | Czechia, distr. Strakonice, Radomyšl: field thicket with Prunus domestica 1.30 km SSE (azimuth 157°) of the church in the village center |
| 055/17 |  |  | 1 |  | 49.11265 | 13.78206 | 08.05.2017 | Czechia, distr. Prachatice, Onšovice: edge of a crop field 490 m ENE (azimuth 70°) of the chapel in the village center |
| 008/18* |  |  | 1 | 1 | 49.14684 | 14.75391 | 19.04.2018 | Czechia, distr. Tábor, Val: edge of a crop field at the NW edge of the village |
| 010/18* | 1 |  | 1 | 1 | 50.00895 | 15.56954 | 20.04.2018 | Czechia, distr. Pardubice, Mokošín: edge of a crop field S of the village |
| 011/18 | 1 |  | 1 |  | 49.91643 | 14.03121 | 26.04.2018 | Czechia, distr. Beroun, Tmaň: edge of a crop field at the bus station "Tmaň, Slavíky, rozc.1.0" |
| 013/18 | 1 |  |  |  | 49.92488 | 14.09110 | 26.04.2018 | Czechia, distr. Beroun, Koněprusy: edge of a crop field S of the Tobolský vrch Hill |
| 014/18 | 1 |  | 1 | 1 | 49.92410 | 14.10618 | 26.04.2018 | Czechia, distr. Beroun, Korno: edge of a crop field at the bus station "Měňany, odb.Koda" |
| 015/18 | 1 |  | 1 | 1 | 49.92236 | 14.14086 | 26.04.2018 | Czechia, distr. Beroun, Korno: edge of a crop field NE of the village |
| 016/18 | 1 |  |  |  | 49.92968 | 14.19701 | 26.04.2018 | Czechia, distr. Beroun, Hlásná Třebaň: edge of a crop field N of the village |
| 017/18 |  |  | 1 | 1 | 49.94528 | 14.21626 | 26.04.2018 | Czechia, distr. Beroun, Mořina: edge of a crop field S of the village |
| 024/18 | 1 |  | 1 | 1 | 49.40900 | 13.55094 | 27.04.2018 | Czechia, distr. Klatovy, Nehodiv: edge of a crop field near the former limestone quarry W of the village |
| 028/18 |  |  | 1 | 1 | 49.35658 | 13.48009 | 27.04.2018 | Czechia, distr. Klatovy, Hnačov: wet meadow at the Hnačovský fishpond |
| 036/18 |  |  | 1 | 1 | 49.58012 | 14.34253 | 28.04.2018 | Czechia, distr. Příbram, Petrovice: edge of a crop field between the villages Skoupý and Týnčany |
| 037/18 |  |  | 1 |  | 49.59717 | 14.37916 | 28.04.2018 | Czechia, distr. Příbram, Počepice: under trees at the cemetery in the village |
| 040/18 | 1 |  | 1 | 1 | 49.30321 | 13.72301 | 29.04.2018 | Czechia, distr. Klatovy, Horažďovice: crop field at the NE slope of the Radlín Hill |
| 042/18* | 1 |  | 1 | 1 | 49.20591 | 13.67517 | 29.04.2018 | Czechia, distr. Klatovy, Soběšice: crop field W of the village |
| 043/18 |  |  | 1 | 1 | 49.14594 | 13.90989 | 29.04.2018 | Czechia, distr. Strakonice, Nišovice: under trees at the St. Vojtěch chapelle between Černětice and Račí |
| 044/18 | 1 |  |  | 1 | 49.30790 | 13.96413 | 30.04.2018 | Czechia, distr. Strakonice, Osek: edge of a crop field between Rohozná and Osek |
| 046/18 |  |  | 1 |  | 49.33296 | 14.03133 | 30.04.2018 | Czechia, distr. Písek, Drhovle: edge of a forest W of the village |
| 075/19 |  |  |  | 1 | 49.38871 | 14.35371 | 13.04.2019 | Czechia, distr. Písek, Veselíčko: under trees in the village |
| 086/19 | 11 |  |  | 15 | 49.30781 | 13.96345 | 14.04.2019 | Czechia, distr. Strakonice, Rohozná: crop-field S of an agricultural road at the N edge of the Chlum Hill, NW of the village OLD09992-10016 |
| 087/19 | 31 |  | 2 | 15 | 49.30804 | 13.96353 | 14.04.2019 | Czechia, distr. Strakonice, Rohozná: crop-field N of an agricultural road at the N edge of the Chlum Hill, NW of the village, OLD09948-09991 |
| 088/19 |  |  | 1 | 1 | 49.30725 | 13.96547 | 14.04.2019 | Czechia, distr. Strakonice, Rohozná: dump site N of an agricultural road at the N edge of the Chlum Hill, NW of the village |
| 095/19 | 1 |  | 1 |  | 49.12269 | 13.84576 | 14.04.2019 | Czechia, distr. Prachatice, Čkyně: edge of a crop field NE of the town |

Suppl. Table 6: Results from growth chamber experiment grouped according to species and treatment for Specific Leaf Area, trichome density adaxial and abaxial, trichome length adaxial and abaxial

| Species | Treatment | N | Minimum | Maximum | Mean | Std.dev. |  |
| --- | --- | --- | --- | --- | --- | --- | --- |
| Specific Leaf Area | | | | | | | |
| *V. hederifolia* | A | 24 | 58.89 | 80.18 | 72.40 | 4.39 |  |
|  | B | 24 | 50.32 | 63.25 | 55.83 | 3.61 |  |
|  | C | 12 | 51.68 | 79.49 | 59.47 | 7.62 |  |
|  | D | 20 | 49.84 | 62.96 | 56.39 | 3.80 |  |
|  | E | 24 | 36.62 | 52.95 | 44.01 | 3.84 |  |
| *V. sublobata* | A | 52 | 85.39 | 125.80 | 106.30 | 8.36 |  |
|  | B | 48 | 65.19 | 103.88 | 78.77 | 8.54 |  |
|  | C | 60 | 59.77 | 89.35 | 77.89 | 5.98 |  |
|  | D | 48 | 65.32 | 95.02 | 79.49 | 6.01 |  |
|  | E | 52 | 31.23 | 66.99 | 50.59 | 8.55 |  |
| Trichome density adaxial | | | | | | |  |
| *V. hederifolia* | A | 12 | 1.22 | 2.89 | 1.72 | 0.52 |  |
|  | B | 12 | 0 | 1.89 | 1.04 | 0.44 |  |
|  | C | 6 | 1.67 | 1.67 | 1.31 | 0.30 |  |
|  | D | 10 | 0.56 | 1.89 | 0.94 | 0.39 |  |
|  | E | 12 | 0.56 | 2.22 | 1.08 | 0.42 |  |
| *V. sublobata* | A | 26 | 1.44 | 4.78 | 2.58 | 0.83 |  |
|  | B | 24 | 0.78 | 5.00 | 1.69 | 0.88 |  |
|  | C | 30 | 0.67 | 3.67 | 1.67 | 0.64 |  |
|  | D | 24 | 0.78 | 3.78 | 1.79 | 0.73 |  |
|  | E | 26 | 0.44 | 2.89 | 1.16 | 0.64 |  |
| Trichome density abaxial | | | | | | |  |
| *V. hederifolia* | A | 12 | 0.67 | 4.33 | 1.53 | 0.94 |  |
|  | B | 12 | 0 | 2.22 | 1.40 | 0.62 |  |
|  | C | 6 | 2.56 | 2.56 | 1.69 | 0.59 |  |
|  | D | 10 | 2.67 | 2.67 | 1.48 | 0.64 |  |
|  | E | 12 | 2.22 | 2.22 | 1.38 | 0.45 |  |
| *V. sublobata* | A | 26 | 0.89 | 3.44 | 1.93 | 0.53 |  |
|  | B | 24 | 0.67 | 5.78 | 1.67 | 1.12 |  |
|  | C | 30 | 0.78 | 3.22 | 1.49 | 0.51 |  |
|  | D | 24 | 0.67 | 2.89 | 1.53 | 0.63 |  |
|  | E | 26 | 0.56 | 3.44 | 1.34 | 0.78 |  |
| Trichome length adaxial | | | | | | |  |
| *V. hederifolia* | A | 12 | 0.29 | 0.53 | 0.39 | 0.06 |  |
|  | B | 12 | 0.33 | 0.56 | 0.46 | 0.06 |  |
|  | C | 6 | 0.38 | 0.53 | 0.49 | 0.06 |  |
|  | D | 10 | 0.36 | 0.56 | 0.46 | 0.05 |  |
|  | E | 12 | 0.37 | 0.54 | 0.45 | 0.05 |  |
| *V. sublobata* | A | 26 | 0.15 | 0.38 | 0.25 | 0.05 |  |
|  | B | 24 | 0.19 | 0.50 | 0.29 | 0.08 |  |
|  | C | 30 | 0.20 | 0.38 | 0.27 | 0.05 |  |
|  | D | 24 | 0.20 | 0.39 | 0.27 | 0.06 |  |
|  | E | 26 | 0.20 | 0.51 | 0.28 | 0.06 |  |
| Trichome length abaxial | | | | | | |  |
| *V. hederifolia* | A | 12 | 0.22 | 0.42 | 0.32 | 0.06 |  |
|  | B | 12 | 0.28 | 0.52 | 0.38 | 0.07 |  |
|  | C | 6 | 0.28 | 0.44 | 0.36 | 0.06 |  |
|  | D | 10 | 0.25 | 0.47 | 0.38 | 0.07 |  |
|  | E | 12 | 0.33 | 0.55 | 0.40 | 0.07 |  |
| *V. sublobata* | A | 26 | 0.15 | 0.38 | 0.28 | 0.05 |  |
|  | B | 24 | 0.26 | 0.44 | 0.33 | 0.05 |  |
|  | C | 30 | 0.27 | 0.49 | 0.34 | 0.05 |  |
|  | D | 24 | 0.25 | 0.48 | 0.34 | 0.05 |  |
|  | E | 26 | 0.24 | 0.45 | 0.32 | 0.06 |  |

Suppl. Table 7: Tests for significance of the results from growth chamber experiment grouped according to species and treatment for Specific Leaf Area, trichome density adaxial and abaxial, trichome length adaxial and abaxial

| Species | Pairwise-Wilcoxon | | | | | Kruskall-Wallis |  |
| --- | --- | --- | --- | --- | --- | --- | --- |
| Specific Leaf Area | | | | | | | |
| *V. hederifolia* |  | A | B | C | D | <0.001 |  |
|  | B | <0.001 |  |  |  |  |  |
|  | C | <0.001 | 0.16 |  |  |  |  |
|  | D | <0.001 | 0.54 | 0.35 |  |  |  |
|  | E | <0.001 | <0.001 | <0.001 | <0.001 |  |  |
| *V. sublobata* |  | A | B | C | D | <0.001 |  |
|  | B | <0.001 |  |  |  |  |  |
|  | C | <0.001 | 0.98 |  |  |  |  |
|  | D | <0.001 | 0.42 | 0.31 |  |  |  |
|  | E | <0.001 | <0.001 | <0.001 | <0.001 |  |  |
| Trichome density adaxial | | | | | | |  |
| *V. hederifolia* |  | A | B | C | D | <0.001 |  |
|  | B | <0.001 |  |  |  |  |  |
|  | C | 0.11 | 0.17 |  |  |  |  |
|  | D | <0.001 | 0.18 | 0.04 |  |  |  |
|  | E | <0.001 | 0.86 | 0.12 | 0.26 |  |  |
| *V. sublobata* |  | A | B | C | D | <0.001 |  |
|  | B | <0.001 |  |  |  |  |  |
|  | C | <0.001 | 0.79 |  |  |  |  |
|  | D | <0.001 | 0.38 | 0.43 |  |  |  |
|  | E | <0.001 | <0.01 | <0.001 | <0.001 |  |  |
| Trichome density abaxial | | | | | | |  |
| *V. hederifolia* |  | A | B | C | D | 0.863 |  |
|  | B | 0.82 |  |  |  |  |  |
|  | C | 0.40 | 0.35 |  |  |  |  |
|  | D | 0.95 | 0.97 | 0.55 |  |  |  |
|  | E | 0.98 | 0.68 | 0.28 | 0.97 |  |  |
| *V. sublobata* |  | A | B | C | D | <0.001 |  |
|  | B | <0.001 |  |  |  |  |  |
|  | C | <0.001 | 0.75 |  |  |  |  |
|  | D | 0.02 | 0.89 | 0.98 |  |  |  |
|  | E | <0.001 | 0.16 | 0.02 | 0.12 |  |  |
| Trichome length adaxial | | | | | | |  |
| *V. hederifolia* |  | A | B | C | D | 0.011 |  |
|  | B | <0.01 |  |  |  |  |  |
|  | C | 0.03 | 0.21 |  |  |  |  |
|  | D | <0.01 | 1 | 0.17 |  |  |  |
|  | E | 0.02 | 0.80 | 0.15 | 0.79 |  |  |
| *V. sublobata* |  | A | B | C | D | 0.321 |  |
|  | B | 0.10 |  |  |  |  |  |
|  | C | 0.24 | 0.50 |  |  |  |  |
|  | D | 0.65 | 0.27 | 0.60 |  |  |  |
|  | E | 0.07 | 0.89 | 0.43 | 0.23 |  |  |
| Trichome length abaxial | | | | | | |  |
| *V. hederifolia* |  | A | B | C | D | 0.093 |  |
|  | B | 0.05 |  |  |  |  |  |
|  | C | 0.16 | 0.55 |  |  |  |  |
|  | D | 0.08 | 0.77 | 0.71 |  |  |  |
|  | E | <0.01 | 0.67 | 0.29 | 0.87 |  |  |
| *V. sublobata* |  | A | B | C | D | <0.001 |  |
|  | B | <0.001 |  |  |  |  |  |
|  | C | <0.001 | 0.30 |  |  |  |  |
|  | D | <0.001 | 0.53 | 0.89 |  |  |  |
|  | E | <0.001 | 0.41 | 0.13 | 0.06 |  |  |

Suppl. Table 8: Results from growth chamber experiment grouped according to species and treatment for leaf lobing characters

| Species | Treatment | N | Minimum | Maximum | Mean | Std.dev. |  |
| --- | --- | --- | --- | --- | --- | --- | --- |
| Central lobe length/width ratio | | | | | | | |
| *V. hederifolia* | A | 24 | 0.35 | 0.68 | 0.47 | 0.09 |  |
|  | B | 24 | 0.30 | 0.69 | 0.48 | 0.08 |  |
|  | C | 12 | 0.39 | 0.64 | 0.49 | 0.06 |  |
|  | D | 20 | 0.29 | 0.64 | 0.46 | 0.10 |  |
|  | E | 24 | 0.35 | 0.91 | 0.53 | 0.15 |  |
| *V. sublobata* | A | 52 | 0.35 | 0.66 | 0.52 | 0.07 |  |
|  | B | 48 | 0.29 | 0.61 | 0.44 | 0.07 |  |
|  | C | 60 | 0.27 | 0.64 | 0.46 | 0.08 |  |
|  | D | 48 | 0.32 | 0.73 | 0.48 | 0.10 |  |
|  | E | 52 | 0.33 | 0.89 | 0.51 | 0.10 |  |
| Incision depth | | | | | | |  |
| *V. hederifolia* | A | 24 | 4.36 | 17.74 | 8.00 | 3.22 |  |
|  | B | 24 | 4.34 | 25.14 | 6.99 | 4.15 |  |
|  | C | 12 | 5.24 | 18.04 | 8.24 | 4.47 |  |
|  | D | 20 | 5.04 | 43.77 | 10.49 | 8.77 |  |
|  | E | 24 | 4.23 | 29.54 | 7.19 | 5.14 |  |
| *V. sublobata* | A | 52 | 4.58 | 11.46 | 6.80 | 1.17 |  |
|  | B | 48 | 5.55 | 17.12 | 7.82 | 2.21 |  |
|  | C | 60 | 4.60 | 15.07 | 7.37 | 1.61 |  |
|  | D | 48 | 4.38 | 12.27 | 7.02 | 1.84 |  |
|  | E | 52 | 3.87 | 11.14 | 6.37 | 1.63 |  |
| Number of lobes | | | | | | |  |
| *V. hederifolia* | A | 24 | 2 | 6 | 3.83 | 0.96 |  |
|  | B | 24 | 3 | 6 | 3.62 | 0.92 |  |
|  | C | 12 | 2 | 5 | 3.67 | 1.07 |  |
|  | D | 20 | 2 | 6 | 3.55 | 1.00 |  |
|  | E | 24 | 2 | 7 | 4.42 | 1.18 |  |
| *V. sublobata* | A | 52 | 3 | 7 | 5.23 | 1.18 |  |
|  | B | 48 | 3 | 7 | 5.17 | 0.93 |  |
|  | C | 60 | 3 | 7 | 5.28 | 0.72 |  |
|  | D | 48 | 3 | 7 | 5.35 | 0.96 |  |
|  | E | 52 | 3 | 8 | 5.6 | 0.91 |  |

Suppl. Table 9: Tests for significance of the results from growth chamber experiment grouped according to species and treatment for leaf lobing characters

| Species | Pairwise-Wilcoxon | | | | | Kruskall-Wallis |  |
| --- | --- | --- | --- | --- | --- | --- | --- |
| Central lobe length/width ratio | | | | | | | |
| *V. hederifolia* |  | A | B | C | D | 0.555 |  |
|  | B | 0.81 |  |  |  |  |  |
|  | C | 0.36 | 0.50 |  |  |  |  |
|  | D | 0.74 | 0.41 | 0.16 |  |  |  |
|  | E | 0.29 | 0.51 | 0.91 | 0.18 |  |  |
| *V. sublobata* |  | A | B | C | D | <0.001 |  |
|  | B | <0.001 |  |  |  |  |  |
|  | C | <0.001 | 0.25 |  |  |  |  |
|  | D | <0.001 | 0.15 | 0.67 |  |  |  |
|  | E | 0.38 | <0.001 | <0.001 | <0.001 |  |  |
| Incision depth | | | | | | |  |
| *V. hederifolia* |  | A | B | C | D | 0.045 |  |
|  | B | 0.09 |  |  |  |  |  |
|  | C | 0.66 | 0.20 |  |  |  |  |
|  | D | 0.49 | 0.01 | 0.26 |  |  |  |
|  | E | 0.07 | 0.75 | 0.28 | 0.02 |  |  |
| *V. sublobata* |  | A | B | C | D | <0.001 |  |
|  | B | <0.001 |  |  |  |  |  |
|  | C | <0.01 | 0.58 |  |  |  |  |
|  | D | 0.27 | 0.04 | 0.09 |  |  |  |
|  | E | 0.25 | <0.001 | <0.001 | 0.09 |  |  |
| Number of lobes | | | | | | |  |
| *V. hederifolia* |  | A | B | C | D | 0.045 |  |
|  | B | 0.33 |  |  |  |  |  |
|  | C | 0.62 | 0.96 |  |  |  |  |
|  | D | 0.25 | 0.75 | 0.78 |  |  |  |
|  | E | 0.07 | 0.01 | 0.08 | 0.01 |  |  |
| *V. sublobata* |  | A | B | C | D | 0.419 |  |
|  | B | 0.48 |  |  |  |  |  |
|  | C | 0.62 | 0.67 |  |  |  |  |
|  | D | 0.90 | 0.38 | 0.56 |  |  |  |
|  | E | 0.37 | 0.07 | 0.09 | 0.36 |  |  |

| Species | Leaf position | N | Minimum | Maximum | Mean | Std.dev. |  |
| --- | --- | --- | --- | --- | --- | --- | --- |
| Central lobe length/width ratio | | | | | | | |
| *V. hederifolia* | top | 26 | 0.38 | 0.91 | 0.59 | 0.12 |  |
|  | Upper middle | 26 | 0.35 | 0.63 | 0.48 | 0.07 |  |
|  | Lower middle | 26 | 0.30 | 0.62 | 0.46 | 0.06 |  |
|  | bottom | 26 | 0.29 | 0.64 | 0.41 | 0.07 |  |
| *V. sublobata* | top | 65 | 0.32 | 0.89 | 0.53 | 0.10 |  |
|  | Upper middle | 65 | 0.32 | 0.64 | 0.48 | 0.07 |  |
|  | Lower middle | 65 | 0.30 | 0.69 | 0.46 | 0.08 |  |
|  | bottom | 65 | 0.27 | 0.62 | 0.46 | 0.8 |  |
| Incision depth | | | | | | |  |
| *V. hederifolia* | top | 26 | 4.34 | 9.57 | 6.30 | 1.32 |  |
|  | Upper middle | 26 | 4.36 | 14.72 | 6.76 | 2.22 |  |
|  | Lower middle | 26 | 4.23 | 7.77 | 5.56 | 1.00 |  |
|  | bottom | 26 | 4.68 | 43.77 | 13.73 | 8.43 |  |
| *V. sublobata* | top | 65 | 4.58 | 11.12 | 6.75 | 1.23 |  |
|  | Upper middle | 65 | 3.98 | 11.55 | 7.03 | 1.38 |  |
|  | Lower middle | 65 | 3.87 | 9.86 | 6.13 | 1.13 |  |
|  | bottom | 65 | 4.60 | 17.12 | 8.39 | 2.53 |  |
| Number of lobes | | | | | | |  |
| *V. hederifolia* | top | 26 | 3 | 7 | 4.85 | 1.05 |  |
|  | Upper middle | 26 | 2 | 5 | 3.92 | 0.93 |  |
|  | Lower middle | 26 | 3 | 5 | 3.42 | 0.65 |  |
|  | bottom | 26 | 2 | 5 | 3.15 | 0.73 |  |
| *V. sublobata* | top | 65 | 4 | 8 | 5.54 | 0.69 |  |
|  | Upper middle | 65 | 5 | 7 | 5.60 | 0.68 |  |
|  | Lower middle | 65 | 3 | 8 | 5.48 | 0.94 |  |
|  | bottom | 65 | 3 | 7 | 4.69 | 1.13 |  |

Suppl. Table 10: Tests for significance of the results from growth chamber experiment grouped according to species and position on the plant for leaf lobing characters
